# Emerging therapeutic strategies for mitochondrial DNA-related diseases

**DOI:** 10.1016/j.xcrm.2026.102841

**Published:** 2026-06-02

**Authors:** Rubing Shi, Micol Falabella, Jana Aref, Michael G. Hanna, Michal Minczuk, Carlo Viscomi, Robert D.S. Pitceathly

**Affiliations:** 1 Department of Neuromuscular Diseases, University College London Queen Square Institute of Neurology, London, UK; 2 NHS Highly Specialised Service for Rare Mitochondrial Disorders, Queen Square Centre for Neuromuscular Diseases, The National Hospital for Neurology and Neurosurgery, London, UK; 3 Medical Research Council Mitochondrial Biology Unit, University of Cambridge, Cambridge, UK; 4 Department of Clinical Neurosciences, University of Cambridge, Cambridge, UK; 5 Veneto Institute of Molecular Medicine, Via Orus 2, 35129 Padova, Italy; 6 Department of Biosciences, University of Milano, via Celoria 26, 20133 Milano, Italy

**Keywords:** mitochondrial DNA, primary mitochondrial diseases, gene therapy, mitochondrial replacement therapy

## Abstract

Primary mitochondrial diseases (PMDs) are among the most common inherited metabolic disorders, affecting approximately 1 in 4,300 individuals. They result from pathogenic variants in mitochondrial DNA (mtDNA) or nuclear DNA (nDNA) that disrupt oxidative phosphorylation and lead to multisystem disease. Although advances in genomic testing have significantly improved diagnostic rates in PMDs, effective disease-modifying therapies remain limited.

Therapeutic development increasingly focuses on mtDNA-targeted approaches because mtDNA variants are a major cause of disease and may offer opportunities for targeted intervention. Current strategies include allotopic expression, mitochondria-targeted nucleases, and next-generation base editors, which reduce or correct pathogenic mtDNA variants. Other emerging approaches include pharmacological modulation of heteroplasmy, reproductive techniques such as mitochondrial donation, and therapeutic strategies based on mitochondrial transplantation.

This review summarizes advances in gene editing, pharmacological approaches, and reproductive and mitochondrial transplantation strategies for mtDNA-related PMDs, highlighting progress toward more targeted interventions.

## Graphical abstract

**Figure:**
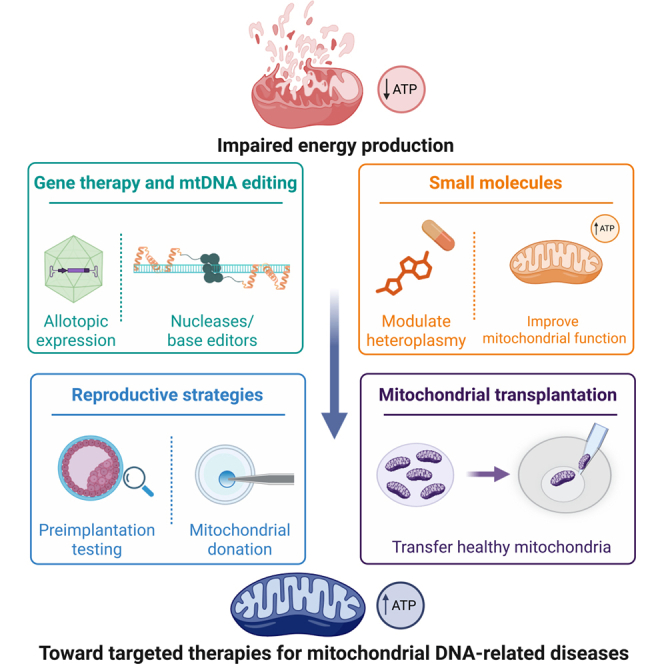

## Teaser

In this review, Shi et al. discuss emerging therapeutic strategies for mitochondrial DNA-related primary mitochondrial diseases, including gene editing, pharmacological modulation of heteroplasmy, mitochondrial donation, and mitochondrial transplantation. It highlights recent progress toward targeted interventions for disorders that currently have limited disease-modifying treatment options.

## Introduction

Primary mitochondrial diseases (PMDs) are among the most common inherited metabolic disorders, affecting approximately 1 in 4,300 individuals.^1^^,^^2^ They are caused by pathogenic variants in either mitochondrial DNA (mtDNA) or nuclear DNA (nDNA), resulting in impaired oxidative phosphorylation (OXPHOS) and diverse, multisystemic clinical manifestations.^3^ PMD-associated mtDNA defects commonly include point mutations and large-scale deletions. Point mutations are frequently maternally inherited, whereas large-scale deletions are typically sporadic and arise *de novo*.^4^ To date, nearly 400 genes have been linked to PMD,^5^^,^^6^ with mtDNA variants accounting for most adult-onset cases (∼80%) and nDNA variants predominating in childhood-onset disease (70%–75%); single large-scale mtDNA deletions are an important exception and often occur in childhood.^3^ This dual genomic origin, together with genetic diversity, creates major challenges for diagnosis, development of effective disease-modifying therapies, and clinical trial design. Despite advances in genetic testing, no approved disease-modifying therapies exist for most PMDs,^3^^,^^7^ and management is largely supportive.

**Figure 1:**
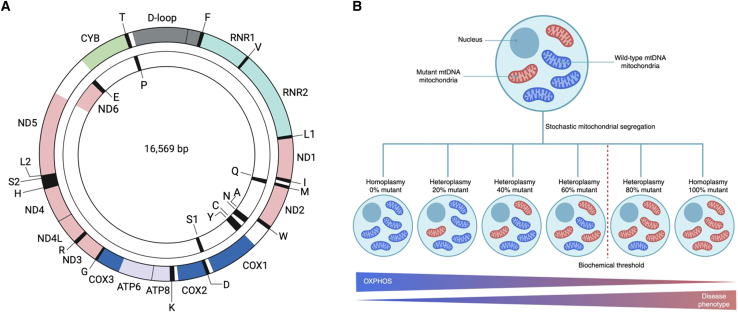
Human mitochondrial genome organization and the heteroplasmy threshold effect (A) Human mitochondrial DNA (mtDNA) is a 16,569-bp circular double-stranded genome located within the mitochondrial matrix and packaged into nucleoprotein complexes known as nucleoids. The mtDNA encodes 13 essential protein subunits of the oxidative phosphorylation (OXPHOS) system (colored segments), 22 transfer RNAs (black), and 2 ribosomal RNAs (light blue). Protein-coding genes are color coded according to their corresponding OXPHOS complexes: complex I (NADH dehydrogenase; ND1–ND6, ND4L; pink), complex III (cytochrome *b*, CYB, green), complex IV (cytochrome *c* oxidase; COX1–COX3; blue), and complex V (ATP synthase; ATP6, ATP8; purple). The non-coding displacement loop (D-loop, gray) contains the origin of replication and regulatory sequences that control mtDNA transcription and replication. (B) Cells can contain a mixture of wild-type (blue) and mutant (red) mitochondrial DNA (mtDNA), a condition known as heteroplasmy. During cell division, mtDNA molecules segregate stochastically, generating daughter cells with varying levels of heteroplasmy, ranging from homoplasmic wildtype (0% mutant) to homoplasmic (100% mutant). Despite the presence of mutant genomes, mitochondrial function is typically preserved until the proportion of mutant mtDNA exceeds a critical biochemical ‘threshold” (often ∼80% but tissue- and mutation dependent), beyond which oxidative phosphorylation (OXPHOS) becomes impaired, leading to biochemical dysfunction and disease phenotype. The figure was created using BioRender.com.

Human mtDNA is a 16,569-bp double-stranded circular genome encoding 37 essential genes required for OXPHOS and mitochondrial protein synthesis^8^ (Figure 1A). It is maternally inherited and present in hundreds to thousands of copies per cell, ranging from ∼100 copies in whole blood to ∼6,000 copies in the heart. These differences largely reflect variation in mitochondrial abundance and energy demand across tissues and cell types^9^ and may also contribute to increased vulnerability to mitochondrial dysfunction. mtDNA can exist in a heteroplasmic state, in which mutant and wild-type genomes coexist within the same cell (Figure 1B). During cell division and inheritance, mtDNA is transmitted stochastically, and the proportion of mutant genomes can vary between cells and tissues. Disease usually develops once the mutant mtDNA level exceeds a threshold that depends on both the variant and the affected tissue. Because heteroplasmy levels can vary across tissues and individuals, clinical presentations can differ, even among family members carrying the same variant. Notably, nearly all mtDNA genes have been associated with PMDs.^10^

Organs with high energy demand, such as the brain, skeletal muscle, and heart, are particularly vulnerable to mitochondrial dysfunction. As a result, PMDs present with a broad spectrum of systemic and neurological features, including fatigue, muscle weakness, seizures, stroke-like episodes, ataxia, hearing and visual loss, and cardiomyopathy.^3^ Disease onset ranges from infancy to adulthood, and severity varies considerably.^3^^,^^11^ The rarity and heterogeneity of PMDs, together with the absence of reliable biomarkers, make diagnosis, mechanistic insight, and clinical trial design particularly challenging.^12^

Over the past decade, substantial progress has been made in developing mtDNA-targeted therapies, particularly for PMDs caused by mtDNA point mutations,^13^^,^^14^^,^^15^ whereas large-scale deletions are generally not amenable to base editing and have been addressed through alternative strategies, such as mitochondrial augmentation of hematopoietic stem cell.^16^ As summarized in Table 1, therapeutic approaches for mtDNA-associated PMDs vary by mutation type and clinical presentation. Targeted nucleases, including mitoTALENs, mtZFNs, and mitoARCUS, and next-generation precision base editors, including DdCBEs, TALEDs, and mitoBE, have demonstrated proof-of-principle in preclinical studies, either by reducing mutant mtDNA load or correcting pathogenic variants. In 2025, studies describing improved delivery systems and next-generation editors have transformed mtDNA gene therapy into an area of tangible translational progress.^17^^,^^18^ Other approaches include (1) allotopic expression of MT-ND4 for Leber hereditary optic neuropathy (LHON; lenadogene nolparvovec/LUMEVOQ), which has advanced to phase III clinical trial evaluation with encouraging, albeit variable, results^19^^,^^20^; (2) small molecules aimed at modulating heteroplasmy; and (3) preventive reproductive approaches, such as mitochondrial donation as well as therapeutic approaches such as mitochondrial transplantation. Despite this progress, heteroplasmy, maternal inheritance, and the limited ability of mitochondria to import or repair nucleic acids continue to slow progress toward safe and effective therapies. Furthermore, the scarcity of robust animal models, small patient populations, and ethical and regulatory challenges remain major barriers to clinical translation.

**Table 1:** Clinical features of mtDNA-related diseases with therapeutic strategies

Disease	Gene (variant)	Clinical symptoms	Approach
**Childhood onset**			

LS	MT-ND3 (m.10191T>C)	progressive neurodegeneration, DD and regression, hypotonia, ataxia, dystonia, with brainstem and basal ganglia involvement, lactic acidosis^21^	BBR, RHPS4^22^
	MT-ND5 (m.13513G>A)		mitoTALENs^23^^,^^24^^,^^25^
	MT-ATP6 (m.8993T>G)		mitoREs^26^^,^^27^; mtZFNs^28^^,^^29^^,^^30^^,^^31^
MS	MT-CO1 (m.6930G>A)	bilateral cataracts, progressive SNHL, myoclonic epilepsy, ataxia, MW, optic atrophy with visual loss, and severe sensorimotor neuropathy^32^	CDDO^33^
PMPS	SLSMD (m.8483_13459del)	part of the spectrum of mtDNA deletion syndrome; refractory sideroblastic anemia with vacuolization of marrow precursors and exocrine pancreatic dysfunction with lactic acidosis, pancreatic insufficiency, renal tubulopathy^34^^,^^35^	mtZFNs^29^; mitoTALENs^36^; CDDO^33^

**Adult onset (spectrum)**			

MELAS	MT-TL1 (m.3243A>G)	stroke-like episodes, encephalopathy, myopathy, seizures, migraine-like headaches, lactic acidosis, hearing loss, cortical blindness, MW^37^^,^^38^	mitoARCUS^39^; mitoTALENs^40^^,^^41^; LY294002, GDC0941, MK2206, rapamycin^42^^,^^43^; 2DG,5TG^44^
	MT-ND5 (m.13513G>A)		mitoTALENs^23^^,^^24^^,^^25^
CM	MT-TA (m.5024C>T)	preclinical models exhibit mild, age-dependent cardiomyopathy^45^	mitoZFN^46^^,^^47^ mitoTALENs^48^ mitoARCUS^49^ DdCBEs^50^
	MT-TI (m.4300A>G)	HCM with myocardial fibrosis, ventricular arrhythmias^51^	DdCBEs^52^
LHON	MT-ND4 (m.11778G>A)	bilateral visual loss and potential multiple sclerosis-like symptoms, movement disorders^53^	allotopic expression (GS010)^19^^,^^20^^,^^54^^,^^55^^,^^56^^,^^57^^,^^58^^,^^59^^,^^60^^,^^61^; mitoABE^62^; TALEDs^63^; rapamycin^64^
	MT-ND6 (m.14459G>A)		mitoTALENs^36^^,^^65^
	MT-ND6 (m.14484T>C)		allotopic expression^66^
NARP	MT-ATP6 (m.9176T>C)	usually childhood onset, with adult onset reported, part of the MT-ATP6 disease spectrum with MILS,^67^ MW, sensory axonal neuropathy, cerebellar ataxia, and retinitis pigmentosa^68^	mitoTALENs^65^
	MT-ATP6 (m.8993T>G)		mitoREs^26^^,^^27^; mtZFNs^28^^,^^29^^,^^30^^,^^31^
KSS	SLSMD (m.8483_13459del; m.5835_9753del, m.12113_14421del, m.8470_13447del, m.7194_14959del, m.9102_15815del)	part of the mtDNA deletion syndrome spectrum; onset before age 20, CPEO, pigmentary retinopathy, cardiac conduction defects, ataxia, hearing loss, growth deficiency, cognitive impairment, tremor, CM.^69^	mtZFNs^29^; mitoTALENs^36^; CDDO^33^; mitochondrial augmentation^16^
CPEO		part of the mtDNA deletion syndrome spectrum; ptosis, ophthalmoplegia, myopathy, dysphagia, exercise intolerance, neuropathy diabetes mellitus and/or optic neuropathy^69^	
MERRF	MT-TK (m.8344A>G)	MERRF and cerebellar ataxia with multisystem involvement including pigmentary retinopathy, lactic acidosis, multiple lipomatosis^70^	mitoTALENs^23^; mitoTev-TALE^71^
GLS	MT-TI (m.4291T>C)	renal tubular salt wasting, hypokalemic alkalosis, hypomagnesemia, hypocalciuria^72^	DdCBEs^17^

**Abbreviations:** 2DG, 2-deoxy-D-glucose; 5TG, 5-thioglucose; BBR, berberine hydrochloride; CDDO, 2-cyano-3,12-dioxo-oleana-1,9(11)-dien-28-oic acid; CM, cardiomyopathy; CPEO, chronic progressive external ophthalmoplegia; DD, developmental delay; DdCBEs, DddA-derived cytosine base editors; GLS, Gitelman-like syndrome; HCM, hypertrophic cardiomyopathy; KSS, Kearns-Sayre syndrome; LHON, Leber hereditary optic neuropathy; LS, Leigh syndrome; MELAS, mitochondrial encephalomyopathy, lactic acidosis and stroke-like episodes; MERRF, myoclonic epilepsy with ragged red fibers; mitoABE, mitochondria-targeted adenine base editor; mitoREs, mitochondria-targeted restriction endonucleases; MILS, maternally inherited Leigh syndrome; mitoTALENs, mitochondria-targeted transcription activator–like effector nucleases; MS, multisystem mitochondrial disorder; MT-ATP6, mitochondrially encoded ATP synthase membrane subunit 6; MT-CO1, mitochondrially encoded cytochrome *c* oxidase subunit 1; mtDNA, mitochondrial DNA; MT-ND3/4/5/6, mitochondrially encoded NADH dehydrogenase subunits 3, 4, 5, and 6; MT-TA, mitochondrially encoded tRNA-Ala; MT-TI, mitochondrially encoded tRNA-Ile; MT-TK, mitochondrially encoded tRNA-Lys; MT-TL1, mitochondrially encoded tRNA leucine 1; mtZFNs, mitochondria-targeted zinc finger nucleases; MW, muscle weakness; NARP, neuropathy, ataxia, and retinitis pigmentosa; PMPS, Pearson marrow-pancreas syndrome; RHPS4, 3,11-Difluoro-6,8,13-trimethyl-8H-quino[4,3,2-kL] acridinium methylsulfate; SNHL, sensorineural hearing loss; SLSMD, single large-scale mitochondrial DNA deletion syndrome; TALEDs, TALE-linked deaminases.

In this review, we focus on emerging therapeutic strategies targeting mtDNA-driven PMDs. We discuss gene therapy approaches, including allotopic expression, programmable nucleases, and precision base editing, as well as pharmacological interventions, preventive reproductive strategies, and mitochondrial transplantation. Together, these approaches highlight the opportunities and current challenges in the mtDNA field, as well as the steps required to advance toward clinical application.

## Biological barriers to mtDNA gene therapy

Targeting mtDNA for therapy presents distinct challenges. Its multicopy nature and heteroplasmy complicate both disease expression and therapeutic efficacy.^73^ In addition, the physical organization of mitochondria restricts access to nucleic acids and limits the delivery of therapeutic molecules.^74^ These barriers continue to create significant challenges to the development of effective mtDNA-based therapies.

A characteristic feature of mtDNA-related disease is heteroplasmy, the coexistence of mutant and wild-type mtDNA within the same cell. In some cases, pathogenic mtDNA variants may be present in all mitochondrial genomes, a state known as homoplasmy.^73^ Clinical and biochemical dysfunction generally emerges when the mutant load exceeds a mutation- and tissue-specific threshold. In skeletal muscles, for example, respiratory-deficient fibers typically harbor high levels of mutant mtDNA, often exceeding 80%, although this threshold varies across genotypes, tissues, and individuals.^75^^,^^76^ For example, individuals from the same family carrying the MELAS-associated m.13045A>G variant can present with different levels of mutation load in skeletal muscle, while levels in other tissues, such as blood and urine,^77^ are very low or undetectable. Reported symptomatic thresholds may be lower for some rare mtDNA point mutations,^78^ although the supporting evidence remains limited. Thresholds are also highly variable for large-scale deletions, with mutation loads in skeletal muscle ranging from 9% to 90%.^79^^,^^80^ Heteroplasmy is also dynamic, shifting over time in somatic tissues and during maternal transmission^75^^,^^81^ (Figure 1B). For example, in patients with the common m.3243A>G mutation, heteroplasmy levels in blood, urine, and skeletal muscle correlate with disease burden; however, tissue variability complicates the prediction of severity.^82^ Notably, not all mtDNA disorders are heteroplasmic. In LHON, for example, most affected individuals carry homoplasmic mutations, including the most common variant, m.11778G>A. Accordingly, therapeutic strategies for LHON are more likely to depend on restoring protein function than on shifting heteroplasmy.^83^

The mtDNA is packaged into nucleoprotein complexes, known as nucleoids, within the mitochondrial matrix, which is enclosed by the outer mitochondrial membrane (OMM) and the inner mitochondrial membrane (IMM). The two membranes have different permeabilities, with the OMM being relatively porous and the IMM being highly impermeable, creating a barrier for nucleic acid delivery.^74^ Mitochondria rely on peptide targeting sequences for protein import via the TOM/TIM complexes, but in mammals, no efficient endogenous pathway exists for importing DNA or large RNAs into the matrix.^84^ Limited, tissue-specific import of a few small RNAs has been described, but it is insufficient for therapeutic purposes.^85^^,^^86^ This lack of RNA import has critical implications for gene therapy. As a result, conventional CRISPR-Cas systems cannot be applied in mitochondria, as they require guide RNAs to direct the Cas protein to the target mtDNA in the matrix. However, recent work has shown that peptide-morpholino chimaeras can be trafficked into mitochondria to silence mitochondrial RNAs, suggesting that engineered RNA import routes into the mitochondrial matrix may be achieved through careful design.^87^

These biological barriers have driven the development of RNA-independent tools, including mitochondria-targeted nucleases (mtZFNs, mitoTALENs, mitoARCUS) and next-generation base editors (DdCBEs, TALEDs, mitoBEs).^13^^,^^14^^,^^15^^,^^49^^,^^88^ Preclinical studies in cell and animal models demonstrate that these approaches can selectively eliminate pathogenic mtDNA variants by shifting heteroplasmy in favor of wild-type genomes or, in the case of base editors, can directly correct pathogenic variants to restore mitochondrial protein function. Together, these advances provide proof of principle that the unique barriers of mitochondrial genetics can be overcome.

## Therapeutic strategies for mtDNA diseases

**Figure 2:**
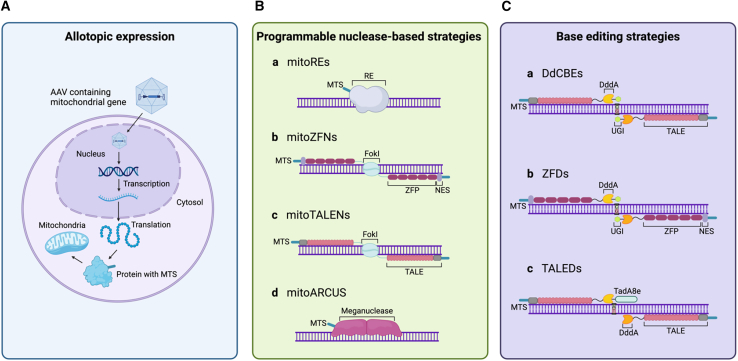
Overview of gene therapy strategies targeting mtDNA-related primary mitochondrial diseases (A) Allotopic expression: nuclear delivery of a recoded mitochondrial gene followed by cytosolic translation and mitochondrial import of the protein via a mitochondrial targeting sequence (MTS). (B) Programmable nuclease-based strategies: mitochondria-targeted restriction endonucleases (mitoREs), zinc-finger nucleases (mitoZFNs), transcription activator-like effector nucleases (mitoTALENs), and ARCUS meganucleases (mitoARCUS) selectively cleave mutant mtDNA to shift heteroplasmy. (C) Base editing strategies: DddA-derived cytosine base editors (DdCBEs), zinc-finger deaminases (ZFDs), and TALE-linked deaminases (TALEDs) enable precise nucleotide conversion in mtDNA without double-strand breaks. The figure was created using BioRender.com.

Given the biological complexity of mitochondria and mtDNA, a range of therapeutic and preventive strategies has been developed either to restore mitochondrial function by directly modifying mtDNA, shifting heteroplasmy, or functionally compensating for mtDNA defects, as well as to reduce the risk of transmitting pathogenic mtDNA. These approaches can be grouped into three main categories: (1) gene therapy-based strategies, which use mitochondria-targeted nucleases or base editors to selectively eliminate or correct pathogenic variants, or restore protein function via allotopic expression; (2) pharmacological interventions that shift heteroplasmy or promote the replication of wild-type mtDNA; and (3) reproductive and mitochondrial transplantation approaches, including mitochondrial donation to reduce the risk of pathogenic mtDNA transmission and mitochondrial transplantation to reduce disease burden (Figure 2). Together, these strategies represent substantial progress toward therapeutic interventions for PMDs and hold promise for clinical translation, although each presents distinct technical and biological challenges.

### Gene therapy approaches

Among therapeutic strategies for mtDNA diseases, gene therapy approaches are the most advanced. These include allotopic expression, programmable nucleases, and base editors. Each strategy addresses the unique barriers of mtDNA genetics either by replacing defective proteins, selectively eliminating mutant genomes, or directly correcting pathogenic variants. While proof of concept has been demonstrated in preclinical and, in some cases, clinical studies, each approach presents distinct technical and translational challenges. Key preclinical studies of gene therapy for PMDs are summarized in Tables S1 and S2.

### Allotopic expression

Allotopic expression involves expressing a wild-type copy of a mitochondrial gene from the nuclear genome, followed by import of the encoded protein into mitochondria. This approach was applied to the only mtDNA-targeted gene therapy to reach clinical trials, GS010 (Lenadogene Nolparvovec) for LHON, which targets the m.11778G>A mutation in *MT-ND4*.^89^ Because LHON is restricted to optic neuropathy with homoplasmic or high heteroplasmy levels, it is particularly amenable to AAV-based gene replacement strategies.^54^ Clinical trials using rAAV2 delivery via a single unilateral intravitreal injection have reported sustained bilateral visual improvement in LHON patients.^19^^,^^20^^,^^55^^,^^56^^,^^57^^,^^58^^,^^59^^,^^60^^,^^61^ An improvement of at least ∼0.3 logMAR in best-corrected visual acuity (BCVA) over baseline was maintained for up to 5 years of follow-up. The treatment was well tolerated, with mild intraocular inflammation reported primarily within the first 2 years after administration. Preclinical studies in cellular models suggest the feasibility of extending this approach to LHON-associated mutations, such as m.14484T>C in *MT-ND6*.^66^ Despite this progress, the application of allotopic expression still has several limitations, including poor import efficiency of hydrophobic proteins and the need for codon and structural optimization to ensure proper mitochondrial targeting and function.^90^^,^^91^^,^^92^

### Programmable nuclease-based strategies

Unlike nuclear DNA, mtDNA relies mainly on base excision repair and lacks other key repair mechanisms, such as nucleotide excision and mismatch repair. As a result, double-strand breaks (DSBs) often lead to rapid DNA degradation.^93^^,^^94^^,^^95^ This unique property has been exploited to selectively eliminate mutant genomes, reducing the heteroplasmy levels and improving disease-associated phenotypes. A proof of principle was first established using mitochondria-targeted restriction endonucleases (mitoREs) *in vitro*^26^^,^^27^ and *in vivo*^96^^,^^97^; however, their application was limited to pathogenic variants with suitable restriction sites. More versatile platforms were developed, including zinc finger nucleases (mtZFNs), transcription activator-like effector nucleases (mitoTALENs), and the more compact ARCUS meganucleases (mitoARCUS). All have demonstrated heteroplasmy shifting and functional rescue in cell and animal models. Each of these tools relies on a mitochondrial targeting sequence (MTS), a short peptide that directs the nuclease to the mitochondrial matrix where mtDNA resides. However, nuclease-based approaches face challenges of delivery; potential off-target cleavage; and, for larger constructs, the need for dual-vector systems. Although mitoTALENs established important proof of principle, the smaller size of mtZFNs and mitoARCUS, together with their potential for single-vector delivery, may make them more suitable for translational development.

mtZFNs are engineered dimers consisting of a non-specific *FokI* nuclease domain fused to zinc-finger DNA binding modules and a mitochondrial targeting sequence. Upon binding and dimerization at mutant mtDNA sites, they generate DSBs that selectively eliminate mutant genomes and shift heteroplasmy toward the wild type.^28^ mtZFNs have been validated *in vitro*^28^^,^^29^^,^^30^^,^^31^ and *in vivo*,^47^ expanding the options for mtDNA manipulation compared to mitoREs. Their main limitations include potential off-target cleavage due to homodimer formation, partial nuclear localization, and the need for dual-vector delivery. A recent study addressed some of these issues by introducing a tandem ZFN architecture that enabled the packaging of both monomers into a single AAV vector, demonstrating effective heteroplasmy reduction and lower immune responses in a m.5024C>T mouse model.^46^ More recent advances in ZFN engineering^98^^,^^99^^,^^100^ have further improved efficiency and specificity. However, mtZFNs have been complemented by more flexible platforms, with higher adaptability in DNA-binding domains for genome editing.

MitoTALENs share the same heterodimeric structure and *FokI*-based cleavage mechanism of mtZFNs but use TALE DNA-binding domains instead of zinc fingers.^36^ Unlike zinc fingers, which recognize nucleotide triplets, each TALE repeat binds a nucleotide, allowing more flexible and precise design with reduced off-target potential. Both *in vitro*^23^^,^^24^^,^^36^^,^^40^^,^^41^^,^^65^^,^^71^ and *in vivo*^48^^,^^65^ studies have shown that mitoTALENs can selectively reduce pathogenic mtDNA variants, such as m.3243A>G and m.13513G>A, shifting the heteroplasmy and promoting phenotypic recovery.^23^^,^^24^^,^^41^ However, their large size and the heterodimeric structure require dual-vector administration, raising potential concerns regarding delivery efficiency and viral dosage safety. To address these issues, several optimizations have been introduced, including altering the size with monomeric recognition,^92^ modifying the FokI domain,^101^^,^^102^ and enhancing DNA recognition by introducing non-conventional repeat-variable di-residues (ncRVDs) to the TALE scaffold.^24^^,^^25^^,^^41^ Despite these challenges, mitoTALENs remain a valuable tool for mtDNA manipulation in preclinical studies and have laid the foundation for next-generation mitochondrial base editors that offer greater precision and more efficient delivery. Moreover, mRNA-lipid nanoparticle (mRNA-LNP) delivery strategies may help bypass the packaging size limitations of viral vectors, enabling the expression of large or complex mitochondrial genes for therapeutic applications.^103^^,^^104^^,^^105^

mitoARCUS are nucleases derived from the homing endonuclease *I-CreI* and engineered to recognize extended DNA sequences with high specificity.^49^ Similar to mtZFNs and mitoTALENs, mitoARCUS requires MTS for import but offers notable advantages, including a relatively small size (∼1.4 kb) and a monomeric structure that allows efficient delivery via a single viral vector within AAV packaging limits.^39^ MitoARCUS has been validated in m.5024C>T mt-tRNA^Ala^ models, where it reduced mutation load, improved mitochondrial respiration *in vitro*, and restored mt-tRNA^Ala^ levels in mouse liver, without detectable off-targeting events.^49^ In a subsequent study, systemic AAV9 delivery of mitoARCUS in a m.3243A>G xenograft mouse model efficiently eliminated mutant mtDNA while preserving the wild-type genome and avoiding nuclear off-target effects, further supporting its translational potential.^39^ However, the limited number of validated sites remains a significant barrier to broader application and clinical development.^39^

### Base editing strategies

Base editing allows precise modification of specific bases in the mtDNA without introducing DSBs. Given that approximately 90% of known pathogenic mtDNA variants are point mutations (www.mitomap.org), this approach has significant therapeutic potential. Importantly, unlike nuclease-based strategies, which rely on selective cleavage of mutant genomes followed by repopulation by residual wild-type mtDNA, base editing offers a unique advantage for modulating homoplasmic mtDNA mutations.^52^ In these cases, even limited correction may generate a small pool of wild-type genomes that can subsequently expand. Over the past few years, rapid progress in CRISPR-free editing tools, including systems such as DdCBEs,^106^ ZFDs,^107^ TALEDs,^108^ mitoBEs,^62^ and CyDENT,^109^ has expanded the toolkit for mtDNA manipulation.

DdCBEs were the first base editors for human mtDNA. They are based on DddA, a bacterial toxin in *Burkholderia cenocepacia* that catalyzes cytosine deamination in double-stranded DNA, enabling programmable C-to-T conversion.^106^ The original DdCBE consists of two monomers with four major parts, including non-toxic split-DddA_tox_ halves that are only activated when brought together, programmable TALE DNA-binding domains, MTS, and an uracil glycosylase inhibitor (UGI) to reduce base excision repair. Proof-of-concept studies have shown efficient mtDNA base editing in multiple PMD-related mtDNA genes in human cell lines with restored OXPHOS.^106^ Further work showed applications in human embryos,^110^ mouse embryos,^111^^,^^112^^,^^113^ adult/neonatal mouse,^114^ and zebrafish,^115^^,^^116^ including evidence of germline transmission.^111^ Patient-derived iPSCs with homoplasmic m.4300A>G variant^52^ and patient-derived fibroblasts with m.4291T>C variant were successfully corrected, restoring mitochondrial membrane potential.^17^ A more recent report has provided evidence that DdCBEs can correct pathogenic mutations *in vivo*. Rather than directly editing the original mutation, researchers introduced a compensatory m.5081G>A base edit by intravenous AAV9 delivery in a mouse model carrying m.5024C>T mt-tRNA^Ala^ variant. This approach stabilized the secondary structure of mt-tRNA^Ala^, restoring mt-tRNA^Ala^ levels in the heart, with no detectable nuclear off-target edits.^50^ Despite their potential, DdCBEs exhibit targeting preferences and off-target effects at neighboring bases,^106^^,^^117^ as well as nuclear off-target activity,^118^ indicating the need for further optimization. Improvements include monomeric DdCBEs (mDdCBEs) for more efficient delivery;^119^ modified TALE recognition modules;^120^ new deaminases from alternative bacterial sources (mitoCBEs, DdCBE_Ss, TALE-SREs);^113^^,^^121^^,^^122^^,^^123^ generating DddA variants by evolution screening with a ∼4.3-fold efficiency increase;^124^ modulating split DddA_tox_ halves to avoid unwanted assembly;^125^ and replacement of TALE scaffolds with zinc fingers (ZFDs and ZF-DdCBEs),^107^^,^^126^ achieving up to 10-fold improvement in editing efficiency.^126^

TALEDs were developed as the first base editors capable of inducing A-to-G conversion. They combine catalytically impaired DddA domains with TadA8e, an engineered adenosine deaminase derived from *Escherichia coli*.^108^ TALEDs have demonstrated efficient mtDNA editing in human cells^108^^,^^127^^,^^128^^,^^129^ and mouse embryos^128^^,^^129^ and have been used to generate a mouse model of Leigh syndrome.^129^ However, like DdCBEs, TALEDs are prone to bystander edits^108^ and exhibit transcriptome-wide RNA off-target activity.^129^ To overcome these issues, additional engineering of DddA_tox_ or TadA8e has been performed.^127^^,^^128^^,^^129^ A recent report revealed the base excision repair mechanism of TALED editing triggered by DddA and developed an enhanced TALED with engineered TadA8e (eTALED6Rs) by replacing DddA with a high-activity variant DddA6.^124^ These advances significantly reduced bystander edits and off-target DNA mutations compared to earlier generations. Based on these findings, Kim et al. performed intravitreal delivery of AAV-based TALED-V28R to correct m.11778G>A mutation in a DdCBE-generated LHON mouse model.^63^

As DddA targets substrate bases on both DNA strands, it creates undesired mutations. To improve the specificity of mitochondrial base editing, a number of studies have replaced the catalytically inactive DddA domain with DNA nickases to introduce single-strand breaks, allowing strand-selective base conversions, reducing unwanted off-target edits, and improving efficiency. Based on this optimized design, strategies including mitoBEs,^62^^,^^130^ CyDENT,^109^ and eTd-mtABEs^131^ have been developed. mtDNA base editors (mitoBEs) consist of TALE DNA-binding domains fused to nickases (MutH or Nt.BspD6I(C)), adenine or cytosine deaminase (TadA8e or APOBEC1), and UGI. These editors have achieved up to 77% efficiency for A-to-G or C-to-T editing and approximately 20% correction of m.11778G>A in patient-derived cells^62^; an updated version reached 82% editing efficiency through deaminase optimisation.^130^ Cytidine deaminase-exonuclease-nickase-TALE (CyDENT) is an alternative strategy that includes a FokI nickase and an additional exonuclease into the base editor constructs for further editing target exposure.^109^ Engineered mtDNA editors (eTd-mtABEs) optimized this principle using directed evolution for more adenine deaminase TadA variants and exhibited a 145-fold increase in editing efficiency compared to split-DddA_tox_ DdCBEs in rat cells.^131^

## Pharmacological approaches: Small molecules that modulate heteroplasmy

Most drugs developed for PMDs aim to support energy production, often through antioxidants or metabolic cofactors.^2^^,^^132^ In this section, we focus on compounds that directly or indirectly target mutant mtDNA or dysfunctional mitochondria to shift heteroplasmy toward wild-type genomes.^133^ Key small molecules reported to modulate mtDNA heteroplasmy are summarized in Table 2.

**Table 2:** Small pharmacological molecules modulating heteroplasmy shift in PMD-related mutations

Molecule	Gene (variant)	Disease	Model	Mechanism	Results	Reference
BBR	MT-ND3 (m.10191T>C)	LS	fibroblasts	inhibiting DNA replication of mutant mtDNA with quadruplex structure	↓ mutant mtDNA improved mitochondrial respiration	Naeem e al.^22^ 2019
RHPS4	MT-ND3 (m.10191T>C)	LS	fibroblasts	inhibiting DNA replication of mutant mtDNA with quadruplex structure	↓ mutant mtDNA	
CDDO	MT-CO1 (m.6930G>A)	MS	cybrids	inhibiting DNA replication by reducing POLG-mtDNA binding via LONP-1/ATFS-1	↓ mutant mtDNA improved mitochondrial respiration	Yang et al.^33^ 2022
	SLSMD (m.8483_13459del)	CPEO, KSS, PMPS				
Stavudine (d4T)	–	–	*D. melanogaster*	inhibiting mtDNA replication	↓ mutant mtDNA	Palozzi et al.^134^ 2022
PZL-A	POLG variants	POLG-RDs	fibroblasts	activating mtDNA synthesis via restoring POLG for mtDNA replication	stimulated mtDNA repopulation, restored respiration function	Valenzuela et al.^135^ 2025
LY294002 and GDC0941	MT-TL1 (m.3243A>G)	MELAS	cybrids/fibroblasts	inhibiting PI3K–AKT–mTORC1 pathway	↓ mutant mtDNA improved mitochondrial respiration	Chung et al.^42^ 2021, Chung et al.^43^ 2025
MK2206						
Rapamycin						
	MT-ND4 (m.11778G>A)	LHON	cybrids	inhibiting PI3K–AKT–mTORC1 pathway	↓ mutant mtDNA, restored ATP levels	Dai et al.^64^ 2014
Torin 1	–	–	NZB/BL6	inhibiting PI3K–AKT–mTORC1 pathway	heteroplasmy shift	Tostes et al.^136^ 2022
2DG and 5TG	MT-TL1 (m.3243A>G)	MELAS	fibroblasts	restricting glucose utilization and suppressing mutant mtDNA replication	heteroplasmy shift, restored respiration	Pantic et al.^44^ 2021

**Abbreviations:** 2DG, 2-deoxy-D-glucose; 5TG, 5-thioglucose; BBR, berberine hydrochloride; CDDO, 2-cyano-3,12-dioxo-oleana-1,9(11)-dien-28-oic acid; CPEO, chronic progressive external ophthalmoplegia; KSS, Kearns-Sayre syndrome; LHON, Leber hereditary optic neuropathy; LS, Leigh syndrome; MELAS, mitochondrial encephalopathy, lactic acidosis and stroke-like episodes; MS, multisystem mitochondrial disorder; mtDNA, mitochondrial DNA; MT-CO1, mitochondrially encoded cytochrome *c* oxidase subunit 1; MT-ND3/4, mitochondrially encoded NADH dehydrogenase subunits 3 and 4; MT-TL1, mitochondrially encoded tRNA-Leu; NZB/BL6, New Zealand Black × C57BL/6 hybrid mice; PMPS, Pearson’s marrow-pancreas syndrome; POLG-RDs, polymerase gamma-related disorders; RHPS4, 3,11-Difluoro-6,8,13-trimethyl-8H-quino[4,3,2-kL] acridinium methylsulfate; SLSMD, single large-scale mitochondrial DNA deletion syndromes.

### mtDNA replication-related molecules

One potential strategy to induce heteroplasmy shift is to directly interfere with the replication of mutant mtDNA, allowing wild-type mtDNA to outcompete the mutant. Based on this principle, researchers have been exploring unique features of mutant mtDNA or defective mitochondria and targeting the regulators of mtDNA replication. Pathogenic variants can form G-quadruplex (GQ) structures, which have been confirmed in several mutations.^22^ Two GQ-binding agents (GQBAs), berberine hydrochloride (BBR) and RHPS4 (3,11-difluoro-6,8,13-trimethyl-8H-quino[4,3,2-kL]acridinium methylsulfate), have been reported to specifically modulate the m.10191T>C mutation by reducing heteroplasmy levels and restoring OXPHOS activity, in Leigh syndrome patient-derived cells.^22^^,^^137^ Another hallmark of dysfunctional mitochondria is the accumulation of ATFS-1 (ATF5 in mammals), which promotes mutant mtDNA replication by increasing POLG binding. In healthy mitochondria, ATFS-1 is degraded by a mitochondrial protease LONP-1. Interestingly, the inhibition of LONP-1 using CDDO (bardoxolone) has been shown to modulate heteroplasmy levels and restore OXPHOS in both worm and human cybrid cells by increasing ATFS-1 and POLG binding to wild-type mtDNA.^33^ However, because bardoxolone has been tested clinically in diabetic kidney disease rather than PMDs, and heart failure events were reported in that setting, its safety would need careful evaluation before translation to mitochondrial disease.^138^ In flies, stavudine, a thymidine analogue that inhibits DNA polymerase γ-dependent mtDNA replication, has been shown to reduce mutation load in germline cells, highlighting its therapeutic potential for mtDNA-related diseases.^134^ Similarly, the small molecule PZL-A has been reported to upregulate mtDNA copy number, a strategy that can dilute mutant genomes and shift heteroplasmy toward wild-type mtDNA.^135^ To date, molecules targeting mtDNA replication have been tested in limited models and require further validation. Future candidates will need to selectively target mitochondria with minimal toxicity to the rest of the cell.

### Metabolic-related molecules

Mutant mtDNA also drives metabolic rewiring that can be exploited therapeutically.^133^ In m.3243A>G patient-derived cells, constitutive activation of the PI3K-AKT-mTORC1 pathway promotes glucose and glutamine utilization while suppressing mitophagy.^42^ Inhibition of this pathway with LY294002 or GDC0941 (PI3K inhibitors), MK2206 (AKT inhibitor), or rapamycin (mTORC1 inhibitor) progressively lowered mutation load in cybrids and fibroblasts while improving OXPHOS activity.^42^^,^^43^ Among these, rapamycin is particularly promising as it is already approved for clinical use in other indications^139^ and has shown preclinical efficacy *in vitro* in a different mtDNA mutation, m.11778G>A.^64^ GDC0941, also known as pictilisib, was investigated in clinical trials for cancer treatment.^140^^,^^141^^,^^142^ Nevertheless, further validation across other mtDNA mutations is required, as current data suggest that heteroplasmy modulation may be mutation specific.^42^ Other mTOR inhibitors, such as torin 1, have also been shown to modulate heteroplasmy in a heteroplasmic mouse model with a mixed genetic background.^136^

Nutrient availability can also influence heteroplasmy dynamics.^143^^,^^144^ OXPHOS-deficient mitochondria often become glutamine-dependent, and restricting this pathway can reduce mutant replication.^145^ In m.3243A>G mutation patient-derived fibroblasts, glucose analogues 2-deoxy-D-glucose (2DG) and 5-thioglucose (5TG) suppressed mutant replication, reduced heteroplasmy, and restored respiration.^44^ Clinically, 2DG has been investigated in trials for epilepsy (NCT05605301, completed), cancer,^146^ and viral infection^147^ and was approved in India in 2021 for the treatment of COVID-19,^148^ highlighting its translational potential. However, recent preclinical work in an mtDNA-depleted epilepsy model reported neuronal degeneration at high doses, emphasizing the importance of careful dose optimization and rigorous safety evaluation.^149^

## Reproductive strategies and mitochondrial transplantation

In addition to pharmacological approaches, preventive reproductive strategies and mitochondrial transplantation approaches have been developed either to reduce the risk of transmitting pathogenic mtDNA or to restore mitochondrial function.^150^ Maternal inheritance of mtDNA represents a substantial risk for women carrying pathogenic variants at high heteroplasmy or homoplasmy, as these variants can be transmitted to their offspring. This risk is amplified by the mtDNA bottleneck, in which a marked reduction and subsequent amplification of mtDNA during oogenesis can unpredictably alter heteroplasmy and influence transmission risk.^151^^,^^152^

### Preimplantation genetic testing for mitochondrial disease

Because pathogenic mtDNA variants can be transmitted from mother to child, reproductive strategies based on *in vitro* fertilization (IVF) are used in clinical practice to reduce this risk. Preimplantation genetic testing for mitochondrial disease (PGT-M) can be used to identify embryos with low levels of mutant mtDNA heteroplasmy.^153^ However, in cases of high maternal heteroplasmy or homoplasmy, suitable embryos may not always be identified, a challenge further complicated by the mtDNA bottleneck, which can unpredictably alter heteroplasmy levels during transmission. In such cases, mitochondrial donation, also known as mitochondrial replacement therapy (MRT), has been developed as an alternative reproductive approach to reduce the risk of pathogenic mtDNA transmission.

### Mitochondrial donation

The principle of mitochondrial donation is to transfer nuclear genetic material from a maternal oocyte or zygote into an enucleated donor oocyte or zygote containing healthy mitochondria. This produces a healthy embryo with nDNA from the parents and mtDNA from the donor. The main approaches being explored include maternal spindle transfer (MST), pronuclear transfer (PNT), and polar body transfer (PBT), all of which aim to reduce the transmission of pathogenic mtDNA. Although ethical and safety concerns remain, mitochondrial donation has been approved in the UK since 2015 and in Australia since 2022.

Maternal spindle transfer (MST) is performed at the oocyte stage prior to fertilization. In metaphase II oocytes, the maternal spindle-chromosome complex is isolated from the maternal oocyte and transferred into an enucleated donor oocyte containing healthy mitochondria. The reconstructed oocyte is then fertilized with paternal sperm to generate an embryo. MST has been demonstrated in non-human primates,^154^^,^^155^ with successful live births and no detectable adverse effects in long-term follow-up,^156^ and has also been applied in human oocytes.^157^^,^^158^^,^^159^ Since oocytes contain a large amount of mtDNA, it is critical to minimize the amount of cytoplasmic material transferred with the spindle during MST, as even low levels of carryover may result in genetic drift and reversion of the pathogenic variant.^155^^,^^160^ A recent study applying MST in patients with infertility reported that 1 out of 6 newborns showed an increase in heteroplasmy level, from 0.8% mtDNA carryover at the blastocyst stage to 30%–60% after birth, indicating the need for cautious application of MST and further optimisation.^161^ The first child born following MST was reported in 2017 in a case involving a mother carrying the Leigh syndrome-associated m.8993T>G variant; the child had low levels of pathogenic mutation load,^162^ although long-term follow-up data remain limited.

PNT is performed in the zygote stage before syngamy, requiring both fertilization of the maternal and donor oocytes with paternal sperm. In zygotes, there is a transient stage when two pronuclei form but do not fuse together. These pronuclei, carrying parental nDNA, are separated via microsurgery without disturbing cytoplasmic compartments and transferred to a donor zygote by microsurgery. It has been tested in early mouse studies,^163^^,^^164^ in human zygotes,^165^ and in non-human primates.^166^ Subsequent optimization in human zygotes reduced maternal mtDNA carryover levels^167^ and improved survival.^168^ To reduce mtDNA heteroplasmy in PNT, a novel approach was developed by artificially inducing mitophagy to selectively eliminate carryover mtDNA, thereby lowering heteroplasmy in mouse and human embryos.^169^ In 2025, a clinical trial reported eight births after mitochondrial donation by PNT, with mtDNA heteroplasmy undetectable or below the pathogenic threshold at birth. Notably, one child exhibited hyperlipidemia, which had also been observed in the mother during pregnancy^153^^,^^170^; this is not a known feature of the maternal variant m.4300A>G. These findings provide important preclinical and clinical evidence regarding the feasibility and safety of this technology, although long-term follow-up is required.

Polar body transfer (PBT) utilizes the by-products of oocyte meiosis, with polar bodies containing a maternal nuclear genome but only minimal cytoplasm and very few mitochondria.^171^^,^^172^ In PBT, either the first polar body (PB1) is isolated and transferred into an enucleated donor oocyte or the second polar body (PB2) to a zygote, respectively. Based on this rationale, PBT exhibits several advantages over MST and PNT, including minimal mtDNA carryover and stable genotype transmission, as polar bodies contain few mitochondria, without the need for cytoskeleton disruptors in the isolation process.^172^ These unique features were further confirmed in human embryos^173^^,^^174^ and applied in a non-human primate model, with the successful generation of primate offspring by PB1T.^175^ However, because polar bodies are small and fragile, they tend to be degraded during processing, potentially leading to incomplete nuclear genome transfer. Further optimization is therefore required to improve the safety and efficiency of PBT in mitochondrial donation.^176^

### Mitochondrial transplantation

Beyond reproductive interventions, mitochondrial transplantation has emerged as a therapeutic strategy. This strategy builds on the natural ability of mitochondria to move between cells via tunneling nanotubes, gap junctions, extracellular vesicles, and other mechanisms.^177^ As a result, mitochondrial transplantation has been widely investigated across a broad spectrum of diseases affecting multiple organ systems,^178^ although evidence in PMDs remains limited. Two main strategies have been explored: (1) pre-conditioning of cellular products with purified mitochondria before administration, and (2) direct delivery of purified mitochondria. For example, mitochondrial transfer from mesenchymal stromal cells (MSCs) to endothelial cells (ECs) has been shown to be critical for EC engraftment in ischemic conditions and for pre-transplantation into ECs, enhancing their *in vivo* integration.^179^ Mitochondrial augmentation therapy (MAT) for hematopoietic stem cells (HSCs) has shown promising evidence in mtDNA-related PMDs, with efficacy observed in immunocompromised mice^180^ and in a first-in-human trial involving 6 patients with single large-scale mtDNA deletion syndromes (SLSMDs).^16^ However, key challenges remain, including immune responses, limited mitochondrial uptake, and heteroplasmy instability, all of which require long-term safety and efficacy evaluation.^181^

## Conclusions

mtDNA-related PMDs account for most adult-onset cases, but therapeutic progress has been limited by heteroplasmy and the difficulty of mitochondrial import. The rarity of PMDs, together with their clinical heterogeneity, limited biomarkers, and unclear genotype-phenotype correlations, continues to hinder early diagnosis, patient recruitment, and the development of effective preclinical models.

Despite these challenges, the field has made clear progress over the past decade. Allotopic expression for LHON has reached phase III clinical testing, and newer genome-editing tools now allow more precise manipulation of mtDNA for research and potential treatment. Mitochondrial donation has become a realistic reproductive option, with successful live births proving its feasibility, and mitochondrial transplantation is being explored as a potential therapeutic strategy. In parallel, pharmacological therapies have also advanced, and the 2025 NICE recommendation of idebenone for visual impairment in people aged 12 years and older with LHON represents an important clinical milestone in mitochondrial medicine.

These emerging approaches reflect a turning point in mitochondrial medicine. Although challenges related to delivery, safety, immune responses, and ethical considerations remain, continued innovation and cross-disciplinary collaboration offer real hope for patients with mtDNA-related PMDs.

## Acknowledgments

Funding: R.D.S.P. is funded by 10.13039/501100022186The Lily Foundation, Muscular Dystrophy UK (MDUK), and a seedcorn award from the Rosetrees Trust and Stoneygate Foundation. R.D.S.P. and M.F. are supported by a UKRI Medical Research Council Transition Support award (MR/X02363X/1). R.D.S.P., M.F., M.G.H., M.M., and C.V. receive support from a UKRI Medical Research Council award (MC_PC_21046) to establish a National Mouse Genetics Network Mitochondria Cluster (MitoCluster). R.D.S.P. and M.G.H. are supported by UKRI Medical Research Council award UKRI2547 – Finding the Missing Worldwide Causes of Inherited Neuromuscular Diseases. R.D.S.P., R.S., M.F., J.A., M.G.H., M.M., and C.V. are supported by the LifeArc Centre to Treat Mitochondrial Diseases (LAC-TreatMito, G125217). LifeArc is a charity registered in England and Wales under no. 1015243 and in Scotland under no. SC037861. The opinions and interpretations presented are those of the authors and not of LifeArc. C.V. is supported by 10.13039/501100002426Telethon Foundation (GSP24003A), Associazione Luigi Comini Onlus, and PNRR Mission 4, Component 2, Investment 1.4-CN00000041 Spoke 1 funded by the 10.13039/501100000780European Union. The University College London Hospitals/University College London Queen Square Institute of Neurology sequencing facility receives a proportion of funding from the Department of Health’s National Institute for Health Research Biomedical Research Centers funding scheme. The clinical and diagnostic “Rare Mitochondrial Disorders” Service in London is funded by the UK NHS Highly Specialised Commissioners.

## Declaration of interests

M.M. is a co-founder, shareholder, and member of the Scientific Advisory Board of Pretzel Therapeutics, Inc. M.M. is the author of a patent application WO2020188228A1 pertaining to the optimization and delivery of mitochondrial proteins in a single expression vector.

## Supplemental information

Document S1. Tables S1 and S2

Document S2. Article plus supplemental information
