# Supplementary material for: Emerging therapeutic strategies for mitochondrial DNA-related diseases
Source: Cell Rep Med. 2026 Jun 2;7(6):102841. doi: 10.1016/j.xcrm.2026.102841 (PMC13293957; doi:10.1016/j.xcrm.2026.102841)
Supplement: Document S1. Tables S1 and S2 [file mmc1.pdf]

Cell Reports Medicine, Volume *7*

Supplemental information

Emerging therapeutic strategies for mitochondrial DNA-related diseases

Rubing Shi, Micol Falabella, Jana Aref, Michael G. Hanna, Michal Minczuk, Carlo Viscomi, and Robert D.S. Pitceathly

Supplementary Table 1. Preclinical *in vitro* studies of gene therapy for PMDs

Gene (variant)	Disease	Model	Results	Refs
**Allotopic expression**				
MT-ND6 (m.14484T>C)	LHON	cybrids	Complex I restoration	17
**mitoREs**				
MT-ATP6 (m.8993T>G)	NARP, MILS	cybrids	↓ mutant mtDNA, restoration of ATP and membrane potential	64
MT-ATP6 (m.8993T>G)	NARP, MILS	cybrids	↓ mutant mtDNA, improved ATP synthase, ↓ lactate	65
**mtZFNs**				
MT-ATP6 (m.8993T>G)	NARP, MILS	cybrids	↓ mutant mtDNA	19
MT-ATP6 (m.8993T>G)	NARP, MILS	cybrids	↓ mutant mtDNA	20
CD (m.8483_13459del4977)	CPEO, KSS, PMPS	cybrids	↓ mutant mtDNA, restoration of MRF	
MT-ATP6 (m.8993T>G)	NARP, MILS	cybrids	↓ mutant mtDNA, restoration of MRF	69
MT-ATP6 (m.8993T>G)	NARP, MILS	cybrids	↓ mutant mtDNA, restoration of MRF	70
**mitoTALENs**				
SLSMD (m.8483_13459del)	CPEO, KSS, PMPS	cybrids	↓ mtDNA deletions and point mutations	21
MT-ND6 (m.14459G>A)	LHON	cybrids	↓ mtDNA deletions and point mutations	
MT-ND6 (m.14459G>A)	LHON	oocytes	↓ mutant mtDNA	75
MT-ATP6 (m.9176T>C)	NARP	oocytes		
MT-TK (m.8344A>G)	MERRF	cybrids	↓ mutant mtDNA, restoration of MRF	76
MT-ND5 (m.13513G>A)	MELAS, LS	cybrids		
MT-TL1 (m.3243A>G)	MELAS	iPSCs, porcine oocytes	↓ mutant mtDNA, restoration of MRF	77
MT-ND5 (m.13513G>A)	MELAS, LS	iPSCs	↓ mutant mtDNA	79
MT-TL1 (m.3243A>G)	MELAS	iPSCs	↓ mutant mtDNA	80
MT-ND5 (m.13513G>A)	MELAS, LS	iPSCs	Reduction mutant mtDNA	83
**mitoTev-TALE**				
MT-TK (m.8344A>G)	MERRF	cybrids	↓ mutant mtDNA, restoration of MRF	78
**mitoARCUS**				
MT-TA (m.5024C>T)	CM	MEFs	↓ mutant mtDNA	23
MT-TL1 (m.3243A>G)	MELAS	cybrids	↓ mutant mtDNA, improved MRF	24
**DdCBEs**				
MT-TI (m.4291T>C)	Gitelman-like syndrome	fibroblasts	↓ mutant mtDNA, rescued MRF	31
MT-TI (m.4300A>G)	HCM	iPSCs	↓ mutant mtDNA, rescued MRF	87
MT-TA (m.5024C>T)	CM	MEFs	restored mitochondrial tRNA^{Ala} level	26
**mitoABE**				
MT-ND4 (m.11778G>A)	LHON	Patient cells	↓ mutant mtDNA, rescued MRF	29
**TALED**				
MT-ND4 (m.11778G>A)	LHON	MEFs	Mutation recovery ratio ~8%, rescued MRF	109

Table S1. Preclinical *in vitro* studies of gene therapy for PMDs. Related to Table 1.
Abbreviations: ATP, adenosine triphosphate; CM, cardiomyopathy; CPEO, chronic progressive external ophthalmoplegia; DdCBEs, DddA-derived cytosine base editors; HCM, hypertrophic cardiomyopathy; iPSCs, induced pluripotent stem cells; KSS, Kearns-Sayre syndrome; LHON, Leber hereditary optic neuropathy; LS, Leigh syndrome; MEFs, mouse embryonic fibroblasts; MELAS, mitochondrial encephalopathy, lactic acidosis and stroke-like episodes; MERRF, myoclonic epilepsy with ragged red fibres; MILS, maternally inherited Leigh syndrome; mitoABE, mitochondria-targeted adenine base editor; mitoREs, mitochondria-targeted restriction endonucleases; mitoTALENs, mitochondria-targeted transcription activator–like effector nucleases; MRF, mitochondrial respiratory function; MT-ATP6, mitochondrially encoded ATP synthase membrane subunit 6; mtDNA, mitochondrial DNA; MT-ND4/5/6, mitochondrially encoded NADH dehydrogenase subunits 4, 5, and 6; MT-TA, mitochondrially encoded tRNA-Ala; MT-TI, mitochondrially encoded tRNA-Ile; MT-TK, mitochondrially encoded tRNA-Lys; MT-TL1, mitochondrially encoded tRNA-Leu; mtZFN, mitochondria-targeted zinc finger nuclease; NARP, neuropathy, ataxia, and retinitis pigmentosa; PMPS, Pearson's marrow-pancreas syndrome; SLSMD, single large-scale mitochondrial DNA deletion syndromes; TALEDs, TALE-linked deaminases.

Supplementary Table 2. Preclinical *in vivo* studies of gene therapy for PMDs

Gene (variant)	Disease	Model	Vector	AR	Target organ	Results	Refs
**mitoREs**							
-	-	NZB/BALB	rAd5/AAV1/2	IC/IM	Brain, SM	efficient shift in mtDNA heteroplasmy	66
-	-	NZB/BALB	AAV9	IP/TV	SM, heart	efficient shift in mtDNA heteroplasmy	67
**mitoZFNs**							
MT-TA (m.5024C>T)	CM	mouse	AAV9.45	IV	Heart	↓ mutant mtDNA, increased stability of $tRNA^{Ala}$, ↓ glycolysis, improved mitochondrial respiration	71
MT-TA (m.5024C>T)	CM	mouse	AAV9.45/AAV9	IM/IV	SM, heart	↓ mutant mtDNA, increased stability of $tRNA^{Ala}$ with minimized immune responses	68
**mitoTALENs**							
-	-	NZB/BALB embryos	-	-	Brain, SM, heart, liver	efficient shift in mtDNA heteroplasmy	75
MT-TA (m.5024C>T)	CM	mouse	AAV9	IM/IV/IP	SM, heart	↓ mutant mtDNA and restored mitochondrial $tRNA^{Ala}$ level	22
**mitoARCUS**							
MT-TA (m.5024C>T)	CM	mouse	AAV9	RO	SM, heart, liver	↓ mutant mtDNA and restored mitochondrial $tRNA^{Ala}$ level	23
MT-TL1 (m.3243A>G)	MELAS	mouse	AAV9	RO	xenograft	↓ mutant mtDNA	24
**DdCBEs**							
MT-TA (m.5024C>T)	CM	mouse	AAV9	RO	SM, heart	restored mitochondrial tRNAAla level and reversed lactate amounts	26
**TALEDs**							
MT-ND4 (m.11778G>A)	LHON	mouse	AAV2	IVT	eye	↓ mutant mtDNA and restored retinal ganglion cell function	109

Table S2. Preclinical *in vivo* studies of gene therapy for PMDs. Related to Table 1.
Abbreviations: AR, administration route; CM, cardiomyopathy; DdCBEs, DddA-derived cytosine base editors; IC, intracerebral; IM, intramuscular; IP, intraperitoneal; iPSCs, induced pluripotent stem cells; IV, intravenous; IVT, intravitreal; LHON, Leber hereditary optic neuropathy; MELAS, mitochondrial encephalopathy, lactic acidosis and stroke-like episodes; mitoABE, mitochondria-targeted adenine base editor; mitoREs, mitochondria-targeted restriction endonucleases; mitoTALENs, mitochondria-targeted transcription activator–like effector nucleases; mtDNA, mitochondrial DNA; MT-TA, mitochondrially encoded tRNA-Ala; MT-TL1, mitochondrially encoded tRNA-Leu; mtZFN, mitochondria-targeted zinc finger nuclease; NZB/BALB, New Zealand Black × BALB/c hybrid mice RO, retro-orbital; SM, skeletal muscle; TALEDs, TALE-linked deaminases; TV, temporal vein.
