# Supplementary material for: Emerging therapeutic strategies for mitochondrial DNA-related diseases
Source: Cell Rep Med. 2026 Jun 2;7(6):102841. doi: 10.1016/j.xcrm.2026.102841 (PMC13293957; doi:10.1016/j.xcrm.2026.102841)
Supplement: Document S2. Article plus supplemental information [file mmc2.pdf]

Cell Reports Medicine

CellPress
OPEN ACCESS

Review

Emerging therapeutic strategies for mitochondrial DNA-related diseases

Rubing Shi,[1,7] Micol Falabella,[1,7,*] Jana Aref,[1] Michael G. Hanna,[1,2] Michal Minczuk,[3,4] Carlo Viscomi,[5,6] and Robert D.S. Pitceathly[1,2,*]
[1]Department of Neuromuscular Diseases, University College London Queen Square Institute of Neurology, London, UK
[2]NHS Highly Specialised Service for Rare Mitochondrial Disorders, Queen Square Centre for Neuromuscular Diseases, The National Hospital for Neurology and Neurosurgery, London, UK
[3]Medical Research Council Mitochondrial Biology Unit, University of Cambridge, Cambridge, UK
[4]Department of Clinical Neurosciences, University of Cambridge, Cambridge, UK
[5]Veneto Institute of Molecular Medicine, Via Orus 2, 35129 Padova, Italy
[6]Department of Biosciences, University of Milano, via Celoria 26, 20133 Milano, Italy
[7]These authors contributed equally
*Correspondence: m.falabella@ucl.ac.uk (M.F.), r.pitceathly@ucl.ac.uk (R.D.S.P.)
https://doi.org/10.1016/j.xcrm.2026.102841

SUMMARY

Primary mitochondrial diseases (PMDs) are among the most common inherited metabolic disorders, affecting approximately 1 in 4,300 individuals. They result from pathogenic variants in mitochondrial DNA (mtDNA) or nuclear DNA (nDNA) that disrupt oxidative phosphorylation and lead to multisystem disease. Although advances in genomic testing have significantly improved diagnostic rates in PMDs, effective disease-modifying therapies remain limited.

Therapeutic development increasingly focuses on mtDNA-targeted approaches because mtDNA variants are a major cause of disease and may offer opportunities for targeted intervention. Current strategies include allotopic expression, mitochondria-targeted nucleases, and next-generation base editors, which reduce or correct pathogenic mtDNA variants. Other emerging approaches include pharmacological modulation of heteroplasmy, reproductive techniques such as mitochondrial donation, and therapeutic strategies based on mitochondrial transplantation.

This review summarizes advances in gene editing, pharmacological approaches, and reproductive and mitochondrial transplantation strategies for mtDNA-related PMDs, highlighting progress toward more targeted interventions.

INTRODUCTION

Primary mitochondrial diseases (PMDs) are among the most common inherited metabolic disorders, affecting approximately 1 in 4,300 individuals.[1,2] They are caused by pathogenic variants in either mitochondrial DNA (mtDNA) or nuclear DNA (nDNA), resulting in impaired oxidative phosphorylation (OXPHOS) and diverse, multisystemic clinical manifestations.[3] PMD-associated mtDNA defects commonly include point mutations and large-scale deletions. Point mutations are frequently maternally inherited, whereas large-scale deletions are typically sporadic and arise *de novo*.[4] To date, nearly 400 genes have been linked to PMD,[5,6] with mtDNA variants accounting for most adult-onset cases (~80%) and nDNA variants predominating in childhood-onset disease (70%–75%); single large-scale mtDNA deletions are an important exception and often occur in childhood.[3] This dual genomic origin, together with genetic diversity, creates major challenges for diagnosis, development of effective disease-modifying therapies, and clinical trial design. Despite advances in genetic testing, no approved disease-modifying therapies exist for most PMDs,[3,7] and management is largely supportive.

Human mtDNA is a 16,569-bp double-stranded circular genome encoding 37 essential genes required for OXPHOS and mitochondrial protein synthesis[8] (Figure 1A). It is maternally inherited and present in hundreds to thousands of copies per cell, ranging from ~100 copies in whole blood to ~6,000 copies in the heart. These differences largely reflect variation in mitochondrial abundance and energy demand across tissues and cell types[9] and may also contribute to increased vulnerability to mitochondrial dysfunction. mtDNA can exist in a heteroplasmic state, in which mutant and wild-type genomes coexist within the same cell (Figure 1B). During cell division and inheritance, mtDNA is transmitted stochastically, and the proportion of mutant genomes can vary between cells and tissues. Disease usually develops once the mutant mtDNA level exceeds a threshold that depends on both the variant and the affected tissue. Because heteroplasmy levels can vary across tissues and individuals, clinical presentations can differ, even among family members carrying the same variant. Notably, nearly all mtDNA genes have been associated with PMDs.[10]

Organs with high energy demand, such as the brain, skeletal muscle, and heart, are particularly vulnerable to mitochondrial

© 2026 The Authors. Published by Elsevier Inc.
This is an open access article under the CC BY license (http://creativecommons.org/licenses/by/4.0/).

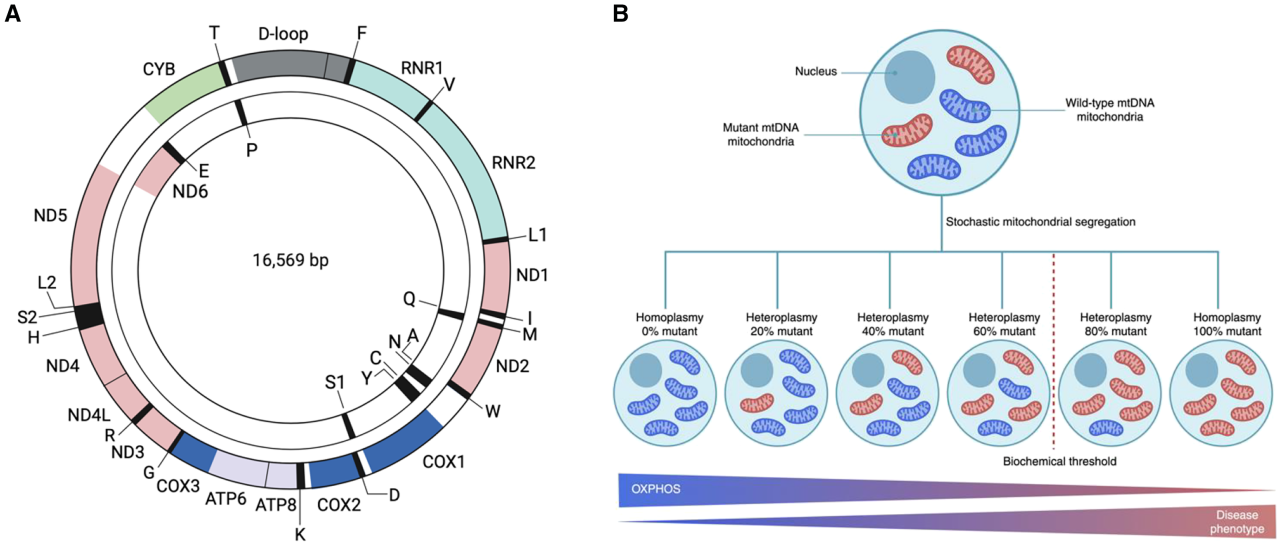

Figure 1. Human mitochondrial genome organization and the heteroplasmy threshold effect

(A) Human mitochondrial DNA (mtDNA) is a 16,569-bp circular double-stranded genome located within the mitochondrial matrix and packaged into nucleoprotein complexes known as nucleoids. The mtDNA encodes 13 essential protein subunits of the oxidative phosphorylation (OXPHOS) system (colored segments), 22 transfer RNAs (black), and 2 ribosomal RNAs (light blue). Protein-coding genes are color coded according to their corresponding OXPHOS complexes: complex I (NADH dehydrogenase; ND1–ND6, ND4L; pink), complex III (cytochrome *b*, CYB, green), complex IV (cytochrome *c* oxidase; COX1–COX3; blue), and complex V (ATP synthase; ATP6, ATP8; purple). The non-coding displacement loop (D-loop, gray) contains the origin of replication and regulatory sequences that control mtDNA transcription and replication.

(B) Cells can contain a mixture of wild-type (blue) and mutant (red) mitochondrial DNA (mtDNA), a condition known as heteroplasmy. During cell division, mtDNA molecules segregate stochastically, generating daughter cells with varying levels of heteroplasmy, ranging from homoplasmic wildtype (0% mutant) to homoplasmic (100% mutant). Despite the presence of mutant genomes, mitochondrial function is typically preserved until the proportion of mutant mtDNA exceeds a critical biochemical 'threshold'' (often ~80% but tissue- and mutation dependent), beyond which oxidative phosphorylation (OXPHOS) becomes impaired, leading to biochemical dysfunction and disease phenotype.

The figure was created using BioRender.com.

dysfunction. As a result, PMDs present with a broad spectrum of systemic and neurological features, including fatigue, muscle weakness, seizures, stroke-like episodes, ataxia, hearing and visual loss, and cardiomyopathy.[3] Disease onset ranges from infancy to adulthood, and severity varies considerably.[3,11] The rarity and heterogeneity of PMDs, together with the absence of reliable biomarkers, make diagnosis, mechanistic insight, and clinical trial design particularly challenging.[12]

Over the past decade, substantial progress has been made in developing mtDNA-targeted therapies, particularly for PMDs caused by mtDNA point mutations,[13–15] whereas large-scale deletions are generally not amenable to base editing and have been addressed through alternative strategies, such as mitochondrial augmentation of hematopoietic stem cell.[16] As summarized in Table 1, therapeutic approaches for mtDNA-associated PMDs vary by mutation type and clinical presentation. Targeted nucleases, including mitoTALENs, mtZFNs, and mitoARCUS, and next-generation precision base editors, including DdCBEs, TALEDs, and mitoBE, have demonstrated proof-of-principle in preclinical studies, either by reducing mutant mtDNA load or correcting pathogenic variants. In 2025, studies describing improved delivery systems and next-generation editors have transformed mtDNA gene therapy into an area of tangible translational progress.[17,18] Other approaches include (1) allotopic expression of MT-ND4 for Leber hereditary optic neuropathy (LHON; lenadogene nolparvovec/LUMEVOQ), which has advanced to phase III clinical trial evaluation with encouraging, albeit variable, results[19,20]; (2) small molecules aimed at modulating heteroplasmy; and (3) preventive reproductive approaches, such as mitochondrial donation as well as therapeutic approaches such as mitochondrial transplantation. Despite this progress, heteroplasmy, maternal inheritance, and the limited ability of mitochondria to import or repair nucleic acids continue to slow progress toward safe and effective therapies. Furthermore, the scarcity of robust animal models, small patient populations, and ethical and regulatory challenges remain major barriers to clinical translation.

In this review, we focus on emerging therapeutic strategies targeting mtDNA-driven PMDs. We discuss gene therapy approaches, including allotopic expression, programmable nucleases, and precision base editing, as well as pharmacological interventions, preventive reproductive strategies, and mitochondrial transplantation. Together, these approaches highlight the opportunities and current challenges in the mtDNA field, as well as the steps required to advance toward clinical application.

BIOLOGICAL BARRIERS TO mtDNA GENE THERAPY

Targeting mtDNA for therapy presents distinct challenges. Its multicopy nature and heteroplasmy complicate both disease expression and therapeutic efficacy.[73] In addition, the physical

Table 1. Clinical features of mtDNA-related diseases with therapeutic strategies

Disease	Gene (variant)	Clinical symptoms	Approach
Childhood onset			
LS	MT-ND3 (m.10191T>C)	progressive neurodegeneration, DD and regression, hypotonia, ataxia, dystonia, with brainstem and basal ganglia involvement, lactic acidosis[21]	BBR, RHPS4[22]
	MT-ND5 (m.13513G>A)		mitoTALENs[23–25]
	MT-ATP6 (m.8993T>G)		mitoREs[26,27]; mtZFNs[28–31]
MS	MT-CO1 (m.6930G>A)	bilateral cataracts, progressive SNHL, myoclonic epilepsy, ataxia, MW, optic atrophy with visual loss, and severe sensorimotor neuropathy[32]	CDDO[33]
PMPS	SLSMD (m.8483_13459del)	part of the spectrum of mtDNA deletion syndrome; refractory sideroblastic anemia with vacuolization of marrow precursors and exocrine pancreatic dysfunction with lactic acidosis, pancreatic insufficiency, renal tubulopathy[34,35]	mtZFNs[29]; mitoTALENs[36]; CDDO[33]
Adult onset (spectrum)			
MELAS	MT-TL1 (m.3243A>G)	stroke-like episodes, encephalopathy, myopathy, seizures, migraine-like headaches, lactic acidosis, hearing loss, cortical blindness, MW[37,38]	mitoARCUS[39]; mitoTALENs[40,41]; LY294002, GDC0941, MK2206, rapamycin[42,43]; 2DG,5TG[44]
	MT-ND5 (m.13513G>A)		mitoTALENs[23–25]
CM	MT-TA (m.5024C>T)	preclinical models exhibit mild, age-dependent cardiomyopathy[45]	mitoZFN[46,47] mitoTALENs[48] mitoARCUS[49] DdCBEs[50]
	MT-TI (m.4300A>G)	HCM with myocardial fibrosis, ventricular arrhythmias[51]	DdCBEs[52]
LHON	MT-ND4 (m.11778G>A)	bilateral visual loss and potential multiple sclerosis-like symptoms, movement disorders[53]	allotopic expression (GS010)[19,20,54–61]; mitoABE[62]; TALEDs[63]; rapamycin[64]
	MT-ND6 (m.14459G>A)		mitoTALENs[36,65]
	MT-ND6 (m.14484T>C)		allotopic expression[66]
NARP	MT-ATP6 (m.9176T>C)	usually childhood onset, with adult onset reported, part of the MT-ATP6 disease spectrum with MILS,[67] MW, sensory axonal neuropathy, cerebellar ataxia, and retinitis pigmentosa[68]	mitoTALENs[65]
	MT-ATP6 (m.8993T>G)		mitoREs[26,27]; mtZFNs[28–31]
KSS	SLSMD (m.8483_13459del; m.5835_9753del, m.12113_14421del, m.8470_13447del, m.7194_14959del, m.9102_15815del)	part of the mtDNA deletion syndrome spectrum; onset before age 20, CPEO, pigmentary retinopathy, cardiac conduction defects, ataxia, hearing loss, growth deficiency, cognitive impairment, tremor, CM.[69]	mtZFNs[29]; mitoTALENs[36]; CDDO[33]; mitochondrial augmentation[16]
CPEO		part of the mtDNA deletion syndrome spectrum; ptosis, ophthalmoplegia, myopathy, dysphagia, exercise intolerance, neuropathy diabetes mellitus and/or optic neuropathy[69]	
MERRF	MT-TK (m.8344A>G)	MERRF and cerebellar ataxia with multisystem involvement including pigmentary retinopathy, lactic acidosis, multiple lipomatosis[70]	mitoTALENs[23]; mitoTev-TALE[71]
GLS	MT-TI (m.4291T>C)	renal tubular salt wasting, hypokalemic alkalosis, hypomagnesemia, hypocalciuria[72]	DdCBEs[17]

Abbreviations: 2DG, 2-deoxy-D-glucose; 5TG, 5-thioglucose; BBR, berberine hydrochloride; CDDO, 2-cyano-3,12-dioxo-oleana-1,9(11)-dien-28-oic acid; CM, cardiomyopathy; CPEO, chronic progressive external ophthalmoplegia; DD, developmental delay; DdCBEs, DddA-derived cytosine base editors; GLS, Gitelman-like syndrome; HCM, hypertrophic cardiomyopathy; KSS, Kearns-Sayre syndrome; LHON, Leber hereditary optic neuropathy; LS, Leigh syndrome; MELAS, mitochondrial encephalomyopathy, lactic acidosis and stroke-like episodes; MERRF, myoclonic epilepsy with ragged red fibers; mitoABE, mitochondria-targeted adenine base editor; mitoREs, mitochondria-targeted restriction endonucleases; MILS, maternally inherited Leigh syndrome; mitoTALENs, mitochondria-targeted transcription activator–like effector nucleases; MS, multisystem mitochondrial disorder; MT-ATP6, mitochondrially encoded ATP synthase membrane subunit 6; MT-CO1, mitochondrially encoded cytochrome *c* oxidase subunit 1; mtDNA, mitochondrial DNA; MT-ND3/4/5/6, mitochondrially encoded NADH dehydrogenase subunits 3, 4, 5, and 6; MT-TA, mitochondrially encoded tRNA-Ala; MT-TI, mitochondrially encoded tRNA-Ile; MT-TK, mitochondrially encoded tRNA-Lys; MT-TL1, mitochondrially encoded tRNA leucine 1; mtZFNs, mitochondria-targeted zinc finger nucleases; MW, muscle weakness; NARP, neuropathy, ataxia, and retinitis pigmentosa; PMPS, Pearson marrow-pancreas syndrome; RHPS4, 3,11-Difluoro-6,8,13-trimethyl-8H-quino[4,3,2-kL] acridinium methylsulfate; SNHL, sensorineural hearing loss; SLSMD, single large-scale mitochondrial DNA deletion syndrome; TALEDs, TALE-linked deaminases.

organization of mitochondria restricts access to nucleic acids and limits the delivery of therapeutic molecules.[74] These barriers continue to create significant challenges to the development of effective mtDNA-based therapies.

A characteristic feature of mtDNA-related disease is heteroplasmy, the coexistence of mutant and wild-type mtDNA within the same cell. In some cases, pathogenic mtDNA variants may be present in all mitochondrial genomes, a state known as homoplasmy.[73] Clinical and biochemical dysfunction generally emerges when the mutant load exceeds a mutation- and tissue-specific threshold. In skeletal muscles, for example, respiratory-deficient fibers typically harbor high levels of mutant mtDNA, often exceeding 80%, although this threshold varies across genotypes, tissues, and individuals.[75,76] For example, individuals from the same family carrying the MELAS-associated m.13045A>G variant can present with different levels of mutation load in skeletal muscle, while levels in other tissues, such as blood and urine,[77] are very low or undetectable. Reported symptomatic thresholds may be lower for some rare mtDNA point mutations,[78] although the supporting evidence remains limited. Thresholds are also highly variable for large-scale deletions, with mutation loads in skeletal muscle ranging from 9% to 90%.[79,80] Heteroplasmy is also dynamic, shifting over time in somatic tissues and during maternal transmission[75,81] (Figure 1B). For example, in patients with the common m.3243A>G mutation, heteroplasmy levels in blood, urine, and skeletal muscle correlate with disease burden; however, tissue variability complicates the prediction of severity.[82] Notably, not all mtDNA disorders are heteroplasmic. In LHON, for example, most affected individuals carry homoplasmic mutations, including the most common variant, m.11778G>A. Accordingly, therapeutic strategies for LHON are more likely to depend on restoring protein function than on shifting heteroplasmy.[83]

The mtDNA is packaged into nucleoprotein complexes, known as nucleoids, within the mitochondrial matrix, which is enclosed by the outer mitochondrial membrane (OMM) and the inner mitochondrial membrane (IMM). The two membranes have different permeabilities, with the OMM being relatively porous and the IMM being highly impermeable, creating a barrier for nucleic acid delivery.[74] Mitochondria rely on peptide targeting sequences for protein import via the TOM/TIM complexes, but in mammals, no efficient endogenous pathway exists for importing DNA or large RNAs into the matrix.[84] Limited, tissue-specific import of a few small RNAs has been described, but it is insufficient for therapeutic purposes.[85,86] This lack of RNA import has critical implications for gene therapy. As a result, conventional CRISPR-Cas systems cannot be applied in mitochondria, as they require guide RNAs to direct the Cas protein to the target mtDNA in the matrix. However, recent work has shown that peptide-morpholino chimaeras can be trafficked into mitochondria to silence mitochondrial RNAs, suggesting that engineered RNA import routes into the mitochondrial matrix may be achieved through careful design.[87]

These biological barriers have driven the development of RNA-independent tools, including mitochondria-targeted nucleases (mtZFNs, mitoTALENs, mitoARCUS) and next-generation base editors (DdCBEs, TALEDs, mitoBEs).[13–15,49,88] Preclinical studies in cell and animal models demonstrate that these approaches can selectively eliminate pathogenic mtDNA variants by shifting heteroplasmy in favor of wild-type genomes or, in the case of base editors, can directly correct pathogenic variants to restore mitochondrial protein function. Together, these advances provide proof of principle that the unique barriers of mitochondrial genetics can be overcome.

THERAPEUTIC STRATEGIES FOR mtDNA DISEASES

Given the biological complexity of mitochondria and mtDNA, a range of therapeutic and preventive strategies has been developed either to restore mitochondrial function by directly modifying mtDNA, shifting heteroplasmy, or functionally compensating for mtDNA defects, as well as to reduce the risk of transmitting pathogenic mtDNA. These approaches can be grouped into three main categories: (1) gene therapy-based strategies, which use mitochondria-targeted nucleases or base editors to selectively eliminate or correct pathogenic variants, or restore protein function via allotopic expression; (2) pharmacological interventions that shift heteroplasmy or promote the replication of wild-type mtDNA; and (3) reproductive and mitochondrial transplantation approaches, including mitochondrial donation to reduce the risk of pathogenic mtDNA transmission and mitochondrial transplantation to reduce disease burden (Figure 2). Together, these strategies represent substantial progress toward therapeutic interventions for PMDs and hold promise for clinical translation, although each presents distinct technical and biological challenges.

Gene therapy approaches

Among therapeutic strategies for mtDNA diseases, gene therapy approaches are the most advanced. These include allotopic expression, programmable nucleases, and base editors. Each strategy addresses the unique barriers of mtDNA genetics either by replacing defective proteins, selectively eliminating mutant genomes, or directly correcting pathogenic variants. While proof of concept has been demonstrated in preclinical and, in some cases, clinical studies, each approach presents distinct technical and translational challenges. Key preclinical studies of gene therapy for PMDs are summarized in Tables S1 and S2.

Allotopic expression

Allotopic expression involves expressing a wild-type copy of a mitochondrial gene from the nuclear genome, followed by import of the encoded protein into mitochondria. This approach was applied to the only mtDNA-targeted gene therapy to reach clinical trials, GS010 (Lenadogene Nolparvovec) for LHON, which targets the m.11778G>A mutation in *MT-ND4*.[89] Because LHON is restricted to optic neuropathy with homoplasmic or high heteroplasmy levels, it is particularly amenable to AAV-based gene replacement strategies.[54] Clinical trials using rAAV2 delivery via a single unilateral intravitreal injection have reported sustained bilateral visual improvement in LHON patients.[19,20,55–61] An improvement of at least ~0.3 logMAR in best-corrected visual acuity (BCVA) over baseline was maintained for up to 5 years of follow-up. The treatment was well tolerated, with mild intraocular inflammation reported primarily within the first 2 years after administration. Preclinical studies in cellular models suggest

CellPress
OPEN ACCESS

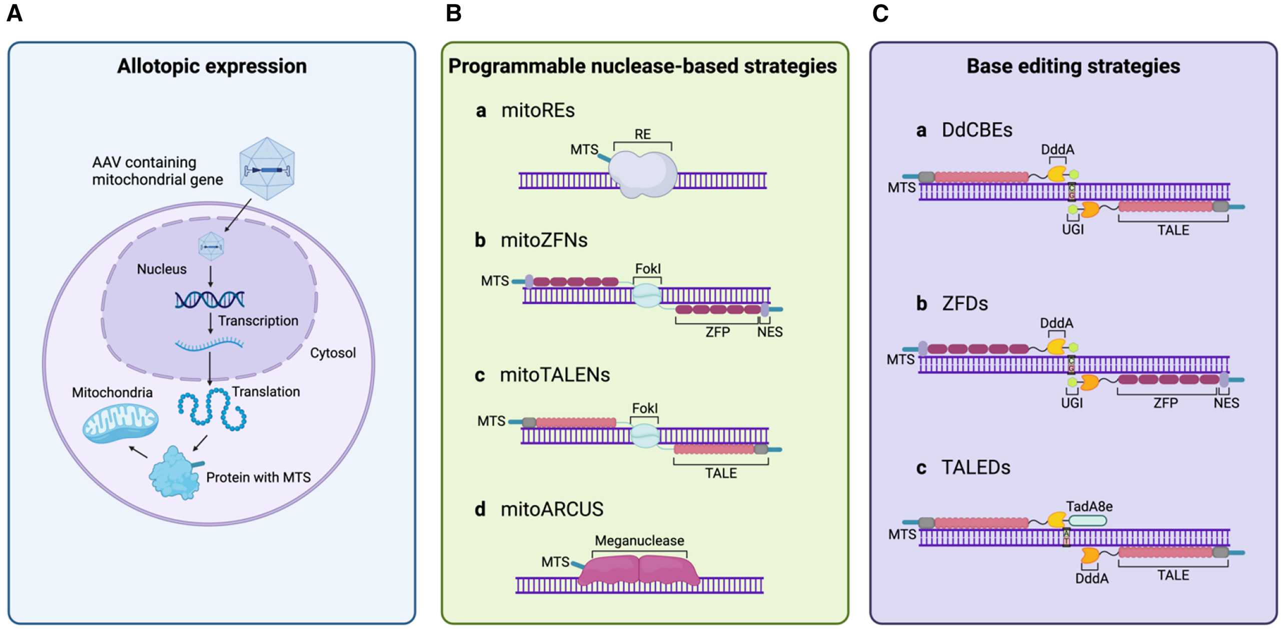

Figure 2. Overview of gene therapy strategies targeting mtDNA-related primary mitochondrial diseases

(A) Allotopic expression: nuclear delivery of a recoded mitochondrial gene followed by cytosolic translation and mitochondrial import of the protein via a mitochondrial targeting sequence (MTS).

(B) Programmable nuclease-based strategies: mitochondria-targeted restriction endonucleases (mitoREs), zinc-finger nucleases (mitoZFNs), transcription activator-like effector nucleases (mitoTALENs), and ARCUS meganucleases (mitoARCUS) selectively cleave mutant mtDNA to shift heteroplasmy.

(C) Base editing strategies: DddA-derived cytosine base editors (DdCBEs), zinc-finger deaminases (ZFDs), and TALE-linked deaminases (TALEDs) enable precise nucleotide conversion in mtDNA without double-strand breaks.

The figure was created using BioRender.com.

the feasibility of extending this approach to LHON-associated mutations, such as m.14484T>C in *MT-ND6*.[66] Despite this progress, the application of allotopic expression still has several limitations, including poor import efficiency of hydrophobic proteins and the need for codon and structural optimization to ensure proper mitochondrial targeting and function.[90–92]

Programmable nuclease-based strategies

Unlike nuclear DNA, mtDNA relies mainly on base excision repair and lacks other key repair mechanisms, such as nucleotide excision and mismatch repair. As a result, double-strand breaks (DSBs) often lead to rapid DNA degradation.[93–95] This unique property has been exploited to selectively eliminate mutant genomes, reducing the heteroplasmy levels and improving disease-associated phenotypes. A proof of principle was first established using mitochondria-targeted restriction endonucleases (mitoREs) *in vitro*[26,27] and *in vivo*[96,97]; however, their application was limited to pathogenic variants with suitable restriction sites. More versatile platforms were developed, including zinc finger nucleases (mtZFNs), transcription activator-like effector nucleases (mitoTALENs), and the more compact ARCUS meganucleases (mitoARCUS). All have demonstrated heteroplasmy shifting and functional rescue in cell and animal models. Each of these tools relies on a mitochondrial targeting sequence (MTS), a short peptide that directs the nuclease to the mitochondrial matrix where mtDNA resides. However, nuclease-based approaches face challenges of delivery; potential off-target cleavage; and, for larger constructs, the need for dual-vector systems. Although mitoTALENs established important proof of principle, the smaller size of mtZFNs and mitoARCUS, together with their potential for single-vector delivery, may make them more suitable for translational development.

mtZFNs are engineered dimers consisting of a non-specific *FokI* nuclease domain fused to zinc-finger DNA binding modules and a mitochondrial targeting sequence. Upon binding and dimerization at mutant mtDNA sites, they generate DSBs that selectively eliminate mutant genomes and shift heteroplasmy toward the wild type.[28] mtZFNs have been validated *in vitro*[28–31] and *in vivo*,[47] expanding the options for mtDNA manipulation compared to mitoREs. Their main limitations include potential off-target cleavage due to homodimer formation, partial nuclear localization, and the need for dual-vector delivery. A recent study addressed some of these issues by introducing a tandem ZFN architecture that enabled the packaging of both monomers into a single AAV vector, demonstrating effective heteroplasmy reduction and lower immune responses in a m.5024C>T mouse model.[46] More recent advances in ZFN engineering[98–100] have further improved efficiency and specificity. However, mtZFNs have been complemented by more flexible platforms, with higher adaptability in DNA-binding domains for genome editing.

MitoTALENs share the same heterodimeric structure and *FokI*-based cleavage mechanism of mtZFNs but use TALE

DNA-binding domains instead of zinc fingers.[36] Unlike zinc fingers, which recognize nucleotide triplets, each TALE repeat binds a nucleotide, allowing more flexible and precise design with reduced off-target potential. Both *in vitro*[23,24,36,40,41,65,71] and *in vivo*[48,65] studies have shown that mitoTALENs can selectively reduce pathogenic mtDNA variants, such as m.3243A>G and m.13513G>A, shifting the heteroplasmy and promoting phenotypic recovery.[23,24,41] However, their large size and the heterodimeric structure require dual-vector administration, raising potential concerns regarding delivery efficiency and viral dosage safety. To address these issues, several optimizations have been introduced, including altering the size with monomeric recognition,[92] modifying the FokI domain,[101,102] and enhancing DNA recognition by introducing non-conventional repeat-variable di-residues (ncRVDs) to the TALE scaffold.[24,25,41] Despite these challenges, mitoTALENs remain a valuable tool for mtDNA manipulation in preclinical studies and have laid the foundation for next-generation mitochondrial base editors that offer greater precision and more efficient delivery. Moreover, mRNA-lipid nanoparticle (mRNA-LNP) delivery strategies may help bypass the packaging size limitations of viral vectors, enabling the expression of large or complex mitochondrial genes for therapeutic applications.[103–105]

mitoARCUS are nucleases derived from the homing endonuclease *I-CreI* and engineered to recognize extended DNA sequences with high specificity.[49] Similar to mtZFNs and mitoTALENs, mitoARCUS requires MTS for import but offers notable advantages, including a relatively small size (~1.4 kb) and a monomeric structure that allows efficient delivery via a single viral vector within AAV packaging limits.[39] MitoARCUS has been validated in m.5024C>T mt-tRNA^{Ala} models, where it reduced mutation load, improved mitochondrial respiration *in vitro*, and restored mt-tRNA^{Ala} levels in mouse liver, without detectable off-targeting events.[49] In a subsequent study, systemic AAV9 delivery of mitoARCUS in a m.3243A>G xenograft mouse model efficiently eliminated mutant mtDNA while preserving the wild-type genome and avoiding nuclear off-target effects, further supporting its translational potential.[39] However, the limited number of validated sites remains a significant barrier to broader application and clinical development.[39]

Base editing strategies

Base editing allows precise modification of specific bases in the mtDNA without introducing DSBs. Given that approximately 90% of known pathogenic mtDNA variants are point mutations (www.mitomap.org), this approach has significant therapeutic potential. Importantly, unlike nuclease-based strategies, which rely on selective cleavage of mutant genomes followed by repopulation by residual wild-type mtDNA, base editing offers a unique advantage for modulating homoplasmic mtDNA mutations.[52] In these cases, even limited correction may generate a small pool of wild-type genomes that can subsequently expand. Over the past few years, rapid progress in CRISPR-free editing tools, including systems such as DdCBEs,[106] ZFDs,[107] TALEDs,[108] mitoBEs,[62] and CyDENT,[109] has expanded the toolkit for mtDNA manipulation.

DdCBEs were the first base editors for human mtDNA. They are based on DddA, a bacterial toxin in *Burkholderia cenocepacia* that catalyzes cytosine deamination in double-stranded DNA, enabling programmable C-to-T conversion.[106] The original DdCBE consists of two monomers with four major parts, including non-toxic split-DddA$_{tox}$ halves that are only activated when brought together, programmable TALE DNA-binding domains, MTS, and an uracil glycosylase inhibitor (UGI) to reduce base excision repair. Proof-of-concept studies have shown efficient mtDNA base editing in multiple PMD-related mtDNA genes in human cell lines with restored OXPHOS.[106] Further work showed applications in human embryos,[110] mouse embryos,[111–113] adult/neonatal mouse,[114] and zebrafish,[115,116] including evidence of germline transmission.[111] Patient-derived iPSCs with homoplasmic m.4300A>G variant[52] and patient-derived fibroblasts with m.4291T>C variant were successfully corrected, restoring mitochondrial membrane potential.[17] A more recent report has provided evidence that DdCBEs can correct pathogenic mutations *in vivo*. Rather than directly editing the original mutation, researchers introduced a compensatory m.5081G>A base edit by intravenous AAV9 delivery in a mouse model carrying m.5024C>T mt-tRNA^{Ala} variant. This approach stabilized the secondary structure of mt-tRNA^{Ala}, restoring mt-tRNA^{Ala} levels in the heart, with no detectable nuclear off-target edits.[50] Despite their potential, DdCBEs exhibit targeting preferences and off-target effects at neighboring bases,[106,117] as well as nuclear off-target activity,[118] indicating the need for further optimization. Improvements include monomeric DdCBEs (mDdCBEs) for more efficient delivery;[119] modified TALE recognition modules;[120] new deaminases from alternative bacterial sources (mitoCBEs, DdCBE_Ss, TALE-SREs);[113,121–123] generating DddA variants by evolution screening with a ~4.3-fold efficiency increase;[124] modulating split DddA$_{tox}$ halves to avoid unwanted assembly;[125] and replacement of TALE scaffolds with zinc fingers (ZFDs and ZF-DdCBEs),[107,126] achieving up to 10-fold improvement in editing efficiency.[126]

TALEDs were developed as the first base editors capable of inducing A-to-G conversion. They combine catalytically impaired DddA domains with TadA8e, an engineered adenosine deaminase derived from *Escherichia coli*.[108] TALEDs have demonstrated efficient mtDNA editing in human cells[108,127–129] and mouse embryos[128,129] and have been used to generate a mouse model of Leigh syndrome.[129] However, like DdCBEs, TALEDs are prone to bystander edits[108] and exhibit transcriptome-wide RNA off-target activity.[129] To overcome these issues, additional engineering of DddA$_{tox}$ or TadA8e has been performed.[127–129] A recent report revealed the base excision repair mechanism of TALED editing triggered by DddA and developed an enhanced TALED with engineered TadA8e (eTALED6Rs) by replacing DddA with a high-activity variant DddA6.[124] These advances significantly reduced bystander edits and off-target DNA mutations compared to earlier generations. Based on these findings, Kim et al. performed intravitreal delivery of AAV-based TALED-V28R to correct m.11778G>A mutation in a DdCBE-generated LHON mouse model.[63]

As DddA targets substrate bases on both DNA strands, it creates undesired mutations. To improve the specificity of mitochondrial base editing, a number of studies have replaced the catalytically inactive DddA domain with DNA nickases to

Table 2. Small pharmacological molecules modulating heteroplasmy shift in PMD-related mutations

Molecule	Gene (variant)	Disease	Model	Mechanism	Results	Reference
BBR	MT-ND3 (m.10191T>C)	LS	fibroblasts	inhibiting DNA replication of mutant mtDNA with quadruplex structure	↓ mutant mtDNA improved mitochondrial respiration	Naeem e al.[22] 2019
RHPS4	MT-ND3 (m.10191T>C)	LS	fibroblasts	inhibiting DNA replication of mutant mtDNA with quadruplex structure	↓ mutant mtDNA	
CDDO	MT-CO1 (m.6930G>A)<br>SLSMD (m.8483_13459del)	MS<br>CPEO, KSS, PMPS	cybrids	inhibiting DNA replication by reducing POLG-mtDNA binding via LONP-1/ATFS-1	↓ mutant mtDNA improved mitochondrial respiration	Yang et al.[33] 2022
Stavudine (d4T)	–	–	*D. melanogaster*	inhibiting mtDNA replication	↓ mutant mtDNA	Palozzi et al.[134] 2022
PZL-A	POLG variants	POLG-RDs	fibroblasts	activating mtDNA synthesis via restoring POLG for mtDNA replication	stimulated mtDNA repopulation, restored respiration function	Valenzuela et al.[135] 2025
LY294002 and GDC0941<br>MK2206<br>Rapamycin	MT-TL1 (m.3243A>G)	MELAS	cybrids/ fibroblasts	inhibiting PI3K–AKT–mTORC1 pathway	↓ mutant mtDNA improved mitochondrial respiration	Chung et al.[42] 2021, Chung et al.[43] 2025
	MT-ND4 (m.11778G>A)	LHON	cybrids	inhibiting PI3K–AKT–mTORC1 pathway	↓ mutant mtDNA, restored ATP levels	Dai et al.[64] 2014
Torin 1	–	–	NZB/BL6	inhibiting PI3K–AKT–mTORC1 pathway	heteroplasmy shift	Tostes et al.[136] 2022
2DG and 5TG	MT-TL1 (m.3243A>G)	MELAS	fibroblasts	restricting glucose utilization and suppressing mutant mtDNA replication	heteroplasmy shift, restored respiration	Pantic et al.[44] 2021

Abbreviations: 2DG, 2-deoxy-D-glucose; 5TG, 5-thioglucose; BBR, berberine hydrochloride; CDDO, 2-cyano-3,12-dioxo-oleana-1,9(11)-dien-28-oic acid; CPEO, chronic progressive external ophthalmoplegia; KSS, Kearns-Sayre syndrome; LHON, Leber hereditary optic neuropathy; LS, Leigh syndrome; MELAS, mitochondrial encephalopathy, lactic acidosis and stroke-like episodes; MS, multisystem mitochondrial disorder; mtDNA, mitochondrial DNA; MT-CO1, mitochondrially encoded cytochrome *c* oxidase subunit 1; MT-ND3/4, mitochondrially encoded NADH dehydrogenase subunits 3 and 4; MT-TL1, mitochondrially encoded tRNA-Leu; NZB/BL6, New Zealand Black × C57BL/6 hybrid mice; PMPS, Pearson's marrow-pancreas syndrome; POLG-RDs, polymerase gamma-related disorders; RHPS4, 3,11-Difluoro-6,8,13-trimethyl-8H-quino[4,3,2-kL] acridinium methylsulfate; SLSMD, single large-scale mitochondrial DNA deletion syndromes.

introduce single-strand breaks, allowing strand-selective base conversions, reducing unwanted off-target edits, and improving efficiency. Based on this optimized design, strategies including mitoBEs,[62,130] CyDENT,[109] and eTd-mtABEs[131] have been developed. mtDNA base editors (mitoBEs) consist of TALE DNA-binding domains fused to nickases (MutH or Nt.BspD6I(C)), adenine or cytosine deaminase (TadA8e or APOBEC1), and UGI. These editors have achieved up to 77% efficiency for A-to-G or C-to-T editing and approximately 20% correction of m.11778G>A in patient-derived cells[62]; an updated version reached 82% editing efficiency through deaminase optimisation.[130] Cytidine deaminase-exonuclease-nickase-TALE (CyDENT) is an alternative strategy that includes a FokI nickase and an additional exonuclease into the base editor constructs for further editing target exposure.[109] Engineered mtDNA editors (eTd-mtABEs) optimized this principle using directed evolution for more adenine deaminase TadA variants and exhibited a 145-fold increase in editing efficiency compared to split-DddA$_{tox}$ DdCBEs in rat cells.[131]

PHARMACOLOGICAL APPROACHES: SMALL MOLECULES THAT MODULATE HETEROPLASMY

Most drugs developed for PMDs aim to support energy production, often through antioxidants or metabolic cofactors.[2,132] In this section, we focus on compounds that directly or indirectly target mutant mtDNA or dysfunctional mitochondria to shift heteroplasmy toward wild-type genomes.[133] Key small molecules reported to modulate mtDNA heteroplasmy are summarized in Table 2.

mtDNA replication-related molecules

One potential strategy to induce heteroplasmy shift is to directly interfere with the replication of mutant mtDNA, allowing wild-type mtDNA to outcompete the mutant. Based on this principle, researchers have been exploring unique features of mutant mtDNA or defective mitochondria and targeting the regulators of mtDNA replication. Pathogenic variants can form G-quadruplex (GQ) structures, which have been confirmed in several mutations.[22]

Two GQ-binding agents (GQBAs), berberine hydrochloride (BBR) and RHPS4 (3,11-difluoro-6,8,13-trimethyl-8H-quino[4,3,2-kL]acridinium methylsulfate), have been reported to specifically modulate the m.10191T>C mutation by reducing heteroplasmy levels and restoring OXPHOS activity, in Leigh syndrome patient-derived cells.[22,137] Another hallmark of dysfunctional mitochondria is the accumulation of ATFS-1 (ATF5 in mammals), which promotes mutant mtDNA replication by increasing POLG binding. In healthy mitochondria, ATFS-1 is degraded by a mitochondrial protease LONP-1. Interestingly, the inhibition of LONP-1 using CDDO (bardoxolone) has been shown to modulate heteroplasmy levels and restore OXPHOS in both worm and human cybrid cells by increasing ATFS-1 and POLG binding to wild-type mtDNA.[33] However, because bardoxolone has been tested clinically in diabetic kidney disease rather than PMDs, and heart failure events were reported in that setting, its safety would need careful evaluation before translation to mitochondrial disease.[138] In flies, stavudine, a thymidine analogue that inhibits DNA polymerase γ-dependent mtDNA replication, has been shown to reduce mutation load in germline cells, highlighting its therapeutic potential for mtDNA-related diseases.[134] Similarly, the small molecule PZL-A has been reported to upregulate mtDNA copy number, a strategy that can dilute mutant genomes and shift heteroplasmy toward wild-type mtDNA.[135] To date, molecules targeting mtDNA replication have been tested in limited models and require further validation. Future candidates will need to selectively target mitochondria with minimal toxicity to the rest of the cell.

Metabolic-related molecules

Mutant mtDNA also drives metabolic rewiring that can be exploited therapeutically.[133] In m.3243A>G patient-derived cells, constitutive activation of the PI3K-AKT-mTORC1 pathway promotes glucose and glutamine utilization while suppressing mitophagy.[42] Inhibition of this pathway with LY294002 or GDC0941 (PI3K inhibitors), MK2206 (AKT inhibitor), or rapamycin (mTORC1 inhibitor) progressively lowered mutation load in cybrids and fibroblasts while improving OXPHOS activity.[42,43] Among these, rapamycin is particularly promising as it is already approved for clinical use in other indications[139] and has shown preclinical efficacy *in vitro* in a different mtDNA mutation, m.11778G>A.[64] GDC0941, also known as pictilisib, was investigated in clinical trials for cancer treatment.[140–142] Nevertheless, further validation across other mtDNA mutations is required, as current data suggest that heteroplasmy modulation may be mutation specific.[42] Other mTOR inhibitors, such as torin 1, have also been shown to modulate heteroplasmy in a heteroplasmic mouse model with a mixed genetic background.[136]

Nutrient availability can also influence heteroplasmy dynamics.[143,144] OXPHOS-deficient mitochondria often become glutamine-dependent, and restricting this pathway can reduce mutant replication.[145] In m.3243A>G mutation patient-derived fibroblasts, glucose analogues 2-deoxy-D-glucose (2DG) and 5-thioglucose (5TG) suppressed mutant replication, reduced heteroplasmy, and restored respiration.[44] Clinically, 2DG has been investigated in trials for epilepsy (NCT05605301, completed), cancer,[146] and viral infection[147] and was approved in India in 2021 for the treatment of COVID-19,[148] highlighting its translational potential. However, recent preclinical work in an mtDNA-depleted epilepsy model reported neuronal degeneration at high doses, emphasizing the importance of careful dose optimization and rigorous safety evaluation.[149]

REPRODUCTIVE STRATEGIES AND MITOCHONDRIAL TRANSPLANTATION

In addition to pharmacological approaches, preventive reproductive strategies and mitochondrial transplantation approaches have been developed either to reduce the risk of transmitting pathogenic mtDNA or to restore mitochondrial function.[150] Maternal inheritance of mtDNA represents a substantial risk for women carrying pathogenic variants at high heteroplasmy or homoplasmy, as these variants can be transmitted to their offspring. This risk is amplified by the mtDNA bottleneck, in which a marked reduction and subsequent amplification of mtDNA during oogenesis can unpredictably alter heteroplasmy and influence transmission risk.[151,152]

Preimplantation genetic testing for mitochondrial disease

Because pathogenic mtDNA variants can be transmitted from mother to child, reproductive strategies based on *in vitro* fertilization (IVF) are used in clinical practice to reduce this risk. Preimplantation genetic testing for mitochondrial disease (PGT-M) can be used to identify embryos with low levels of mutant mtDNA heteroplasmy.[153] However, in cases of high maternal heteroplasmy or homoplasmy, suitable embryos may not always be identified, a challenge further complicated by the mtDNA bottleneck, which can unpredictably alter heteroplasmy levels during transmission. In such cases, mitochondrial donation, also known as mitochondrial replacement therapy (MRT), has been developed as an alternative reproductive approach to reduce the risk of pathogenic mtDNA transmission.

Mitochondrial donation

The principle of mitochondrial donation is to transfer nuclear genetic material from a maternal oocyte or zygote into an enucleated donor oocyte or zygote containing healthy mitochondria. This produces a healthy embryo with nDNA from the parents and mtDNA from the donor. The main approaches being explored include maternal spindle transfer (MST), pronuclear transfer (PNT), and polar body transfer (PBT), all of which aim to reduce the transmission of pathogenic mtDNA. Although ethical and safety concerns remain, mitochondrial donation has been approved in the UK since 2015 and in Australia since 2022.

Maternal spindle transfer (MST) is performed at the oocyte stage prior to fertilization. In metaphase II oocytes, the maternal spindle-chromosome complex is isolated from the maternal oocyte and transferred into an enucleated donor oocyte containing healthy mitochondria. The reconstructed oocyte is then fertilized with paternal sperm to generate an embryo. MST has been demonstrated in non-human primates,[154,155] with successful live births and no detectable adverse effects in long-term follow-up,[156] and has also been applied in human oocytes.[157–159] Since oocytes contain a large amount of mtDNA,

it is critical to minimize the amount of cytoplasmic material transferred with the spindle during MST, as even low levels of carryover may result in genetic drift and reversion of the pathogenic variant.[155,160] A recent study applying MST in patients with infertility reported that 1 out of 6 newborns showed an increase in heteroplasmy level, from 0.8% mtDNA carryover at the blastocyst stage to 30%–60% after birth, indicating the need for cautious application of MST and further optimisation.[161] The first child born following MST was reported in 2017 in a case involving a mother carrying the Leigh syndrome-associated m.8993T>G variant; the child had low levels of pathogenic mutation load,[162] although long-term follow-up data remain limited.

PNT is performed in the zygote stage before syngamy, requiring both fertilization of the maternal and donor oocytes with paternal sperm. In zygotes, there is a transient stage when two pronuclei form but do not fuse together. These pronuclei, carrying parental nDNA, are separated via microsurgery without disturbing cytoplasmic compartments and transferred to a donor zygote by microsurgery. It has been tested in early mouse studies,[163,164] in human zygotes,[165] and in non-human primates.[166] Subsequent optimization in human zygotes reduced maternal mtDNA carryover levels[167] and improved survival.[168] To reduce mtDNA heteroplasmy in PNT, a novel approach was developed by artificially inducing mitophagy to selectively eliminate carryover mtDNA, thereby lowering heteroplasmy in mouse and human embryos.[169] In 2025, a clinical trial reported eight births after mitochondrial donation by PNT, with mtDNA heteroplasmy undetectable or below the pathogenic threshold at birth. Notably, one child exhibited hyperlipidemia, which had also been observed in the mother during pregnancy[153,170]; this is not a known feature of the maternal variant m.4300A>G. These findings provide important preclinical and clinical evidence regarding the feasibility and safety of this technology, although long-term follow-up is required.

Polar body transfer (PBT) utilizes the by-products of oocyte meiosis, with polar bodies containing a maternal nuclear genome but only minimal cytoplasm and very few mitochondria.[171,172] In PBT, either the first polar body (PB1) is isolated and transferred into an enucleated donor oocyte or the second polar body (PB2) to a zygote, respectively. Based on this rationale, PBT exhibits several advantages over MST and PNT, including minimal mtDNA carryover and stable genotype transmission, as polar bodies contain few mitochondria, without the need for cytoskeleton disruptors in the isolation process.[172] These unique features were further confirmed in human embryos[173,174] and applied in a non-human primate model, with the successful generation of primate offspring by PB1T.[175] However, because polar bodies are small and fragile, they tend to be degraded during processing, potentially leading to incomplete nuclear genome transfer. Further optimization is therefore required to improve the safety and efficiency of PBT in mitochondrial donation.[176]

Mitochondrial transplantation

Beyond reproductive interventions, mitochondrial transplantation has emerged as a therapeutic strategy. This strategy builds on the natural ability of mitochondria to move between cells via tunneling nanotubes, gap junctions, extracellular vesicles, and other mechanisms.[177] As a result, mitochondrial transplantation has been widely investigated across a broad spectrum of diseases affecting multiple organ systems,[178] although evidence in PMDs remains limited. Two main strategies have been explored: (1) pre-conditioning of cellular products with purified mitochondria before administration, and (2) direct delivery of purified mitochondria. For example, mitochondrial transfer from mesenchymal stromal cells (MSCs) to endothelial cells (ECs) has been shown to be critical for EC engraftment in ischemic conditions and for pre-transplantation into ECs, enhancing their *in vivo* integration.[179] Mitochondrial augmentation therapy (MAT) for hematopoietic stem cells (HSCs) has shown promising evidence in mtDNA-related PMDs, with efficacy observed in immunocompromised mice[180] and in a first-in-human trial involving 6 patients with single large-scale mtDNA deletion syndromes (SLSMDs).[16] However, key challenges remain, including immune responses, limited mitochondrial uptake, and heteroplasmy instability, all of which require long-term safety and efficacy evaluation.[181]

CONCLUSIONS

mtDNA-related PMDs account for most adult-onset cases, but therapeutic progress has been limited by heteroplasmy and the difficulty of mitochondrial import. The rarity of PMDs, together with their clinical heterogeneity, limited biomarkers, and unclear genotype-phenotype correlations, continues to hinder early diagnosis, patient recruitment, and the development of effective preclinical models.

Despite these challenges, the field has made clear progress over the past decade. Allotopic expression for LHON has reached phase III clinical testing, and newer genome-editing tools now allow more precise manipulation of mtDNA for research and potential treatment. Mitochondrial donation has become a realistic reproductive option, with successful live births proving its feasibility, and mitochondrial transplantation is being explored as a potential therapeutic strategy. In parallel, pharmacological therapies have also advanced, and the 2025 NICE recommendation of idebenone for visual impairment in people aged 12 years and older with LHON represents an important clinical milestone in mitochondrial medicine.

These emerging approaches reflect a turning point in mitochondrial medicine. Although challenges related to delivery, safety, immune responses, and ethical considerations remain, continued innovation and cross-disciplinary collaboration offer real hope for patients with mtDNA-related PMDs.

ACKNOWLEDGMENTS

Funding: R.D.S.P. is funded by The Lily Foundation, Muscular Dystrophy UK (MDUK), and a seedcorn award from the Rosetrees Trust and Stoneygate Foundation. R.D.S.P. and M.F. are supported by a UKRI Medical Research Council Transition Support award (MR/X02363X/1). R.D.S.P., M.F., M.G.H., M.M., and C.V. receive support from a UKRI Medical Research Council award (MC_PC_21046) to establish a National Mouse Genetics Network Mitochondria Cluster (MitoCluster). R.D.S.P. and M.G.H. are supported by UKRI Medical Research Council award UKRI2547 – Finding the Missing Worldwide Causes of Inherited Neuromuscular Diseases. R.D.S.P., R.S., M.F., J.A.,

M.G.H., M.M., and C.V. are supported by the LifeArc Centre to Treat Mitochondrial Diseases (LAC-TreatMito, G125217). LifeArc is a charity registered in England and Wales under no. 1015243 and in Scotland under no. SC037861. The opinions and interpretations presented are those of the authors and not of LifeArc. C.V. is supported by Telethon Foundation (GSP24003A), Associazione Luigi Comini Onlus, and PNRR Mission 4, Component 2, Investment 1.4-CN00000041 Spoke 1 funded by the European Union. The University College London Hospitals/University College London Queen Square Institute of Neurology sequencing facility receives a proportion of funding from the Department of Health's National Institute for Health Research Biomedical Research Centers funding scheme. The clinical and diagnostic "Rare Mitochondrial Disorders" Service in London is funded by the UK NHS Highly Specialised Commissioners.

DECLARATION OF INTERESTS

M.M. is a co-founder, shareholder, and member of the Scientific Advisory Board of Pretzel Therapeutics, Inc. M.M. is the author of a patent application WO2020188228A1 pertaining to the optimization and delivery of mitochondrial proteins in a single expression vector.

SUPPLEMENTAL INFORMATION

Supplemental information can be found online at https://doi.org/10.1016/j.xcrm.2026.102841.

REFERENCES

1. Gorman, G.S., Schaefer, A.M., Ng, Y., Gomez, N., Blakely, E.L., Alston, C.L., Feeney, C., Horvath, R., Yu-Wai-Man, P., Chinnery, P.F., et al. (2015). Prevalence of nuclear and mitochondrial DNA mutations related to adult mitochondrial disease. Ann. Neurol. *77*, 753–759. https://doi.org/10.1002/ana.24362.
2. Wen, H., Deng, H., Li, B., Chen, J., Zhu, J., Zhang, X., Yoshida, S., and Zhou, Y. (2025). Mitochondrial diseases: from molecular mechanisms to therapeutic advances. Signal Transduct. Target. Ther. *10*, 9. https://doi.org/10.1038/s41392-024-02044-3.
3. Gorman, G.S., Chinnery, P.F., DiMauro, S., Hirano, M., Koga, Y., McFarland, R., Suomalainen, A., Thorburn, D.R., Zeviani, M., and Turnbull, D.M. (2016). Mitochondrial diseases. Nat. Rev. Dis. Primers *2*, 16080. https://doi.org/10.1038/nrdp.2016.80.
4. Sallevelt, S.C.E.H., de Die-Smulders, C.E.M., Hendrickx, A.T.M., Hellebrekers, D.M.E.I., de Coo, I.F.M., Alston, C.L., Knowles, C., Taylor, R.W., McFarland, R., and Smeets, H.J.M. (2017). De novo mtDNA point mutations are common and have a low recurrence risk. J. Med. Genet. *54*, 73–83. https://doi.org/10.1136/jmedgenet-2016-103876.
5. Rahman, S. (2020). Mitochondrial disease in children. J. Intern. Med. *287*, 609–633. https://doi.org/10.1111/joim.13054.
6. Heath, O., Feichtinger, R.G., Achleitner, M.T., Hofbauer, P., Mayr, D., Merkevicius, K., Spenger, J., Steinbrücker, K., Steindl, C., Tiefenthaler, E., et al. (2025). Mitochondrial disorder diagnosis and management-what the pediatric neurologist wants to know. Eur. J. Paediatr. Neurol. *54*, 75–88. https://doi.org/10.1016/j.ejpn.2024.10.009.
7. Parikh, S., Goldstein, A., Koenig, M.K., Scaglia, F., Enns, G.M., Saneto, R., Anselm, I., Cohen, B.H., Falk, M.J., Greene, C., et al. (2015). Diagnosis and management of mitochondrial disease: a consensus statement from the Mitochondrial Medicine Society. Genet. Med. *17*, 689–701. https://doi.org/10.1038/gim.2014.177.
8. Anderson, S., Bankier, A.T., Barrell, B.G., de Bruijn, M.H., Coulson, A.R., Drouin, J., Eperon, I.C., Nierlich, D.P., Roe, B.A., Sanger, F., et al. (1981). Sequence and organization of the human mitochondrial genome. Nature *290*, 457–465. https://doi.org/10.1038/290457a0.
9. Rath, S.P., Gupta, R., Todres, E., Wang, H., Jourdain, A.A., Ardlie, K.G., Calvo, S.E., and Mootha, V.K. (2024). Mitochondrial genome copy number variation across tissues in mice and humans. Proc. Natl. Acad. Sci. USA *121*, e2402291121. https://doi.org/10.1073/pnas.2402291121.
10. Schlieben, L.D., and Prokisch, H. (2020). The Dimensions of Primary Mitochondrial Disorders. Front. Cell Dev. Biol. *8*, 600079. https://doi.org/10.3389/fcell.2020.600079.
11. Russell, O.M., Gorman, G.S., Lightowlers, R.N., and Turnbull, D.M. (2020). Mitochondrial Diseases: Hope for the Future. Cell *181*, 168–188. https://doi.org/10.1016/j.cell.2020.02.051.
12. Pizzamiglio, C., Vernon, H.J., Hanna, M.G., and Pitceathly, R.D.S. (2022). Designing clinical trials for rare diseases: unique challenges and opportunities. Nat. Rev. Methods Primers *2*. https://doi.org/10.1038/s43586-022-00100-2.
13. Falabella, M., Minczuk, M., Hanna, M.G., Viscomi, C., and Pitceathly, R.D.S. (2022). Gene therapy for primary mitochondrial diseases: experimental advances and clinical challenges. Nat. Rev. Neurol. *18*, 689–698. https://doi.org/10.1038/s41582-022-00715-9.
14. Phan, H.T.L., Lee, H., and Kim, K. (2023). Trends and prospects in mitochondrial genome editing. Exp. Mol. Med. *55*, 871–878. https://doi.org/10.1038/s12276-023-00973-7.
15. Kim, J.S., and Chen, J. (2024). Base editing of organellar DNA with programmable deaminases. Nat. Rev. Mol. Cell Biol. *25*, 34–45. https://doi.org/10.1038/s41580-023-00663-2.
16. Jacoby, E., Bar-Yosef, O., Gruber, N., Lahav, E., Varda-Bloom, N., Bolkier, Y., Bar, D., Blumkin, M.B.Y., Barak, S., Eisenstein, E., et al. (2022). Mitochondrial augmentation of hematopoietic stem cells in children with single large-scale mitochondrial DNA deletion syndromes. Sci. Transl. Med. *14*, eabo3724. https://doi.org/10.1126/scitranslmed.abo3724.
17. Joore, I.P., Shehata, S., Muffels, I., Castro-Alpízar, J., Jiménez-Curiel, E., Nagyova, E., Levy, N., Tang, Z., Smit, K., Vermeij, W.P., et al. (2025). Correction of pathogenic mitochondrial DNA in patient-derived disease models using mitochondrial base editors. PLoS Biol. *23*, e3003207. https://doi.org/10.1371/journal.pbio.3003207.
18. Norota, K., Ishizuka, S., Hirose, M., Sato, Y., Maeki, M., Tokeshi, M., Ibrahim, S.M., Harashima, H., and Yamada, Y. (2025). Lipid nanoparticle delivery of the CRISPR/Cas9 system directly into the mitochondria of cells carrying m.7778G>T mutation in MtDNA (mt-Atp8). Sci. Rep. *15*, 18717. https://doi.org/10.1038/s41598-025-03671-8.
19. Yu-Wai-Man, P., Newman, N.J., Biousse, V., Carelli, V., Moster, M.L., Vignal-Clermont, C., Klopstock, T., Sadun, A.A., Sergott, R.C., Hage, R., et al. (2025). Five-Year Outcomes of Lenadogene Nolparvovec Gene Therapy in Leber Hereditary Optic Neuropathy. JAMA Ophthalmol. *143*, 99–108. https://doi.org/10.1001/jamaophthalmol.2024.5375.
20. Carelli, V., Newman, N.J., Yu-Wai-Man, P., Biousse, V., Moster, M.L., Subramanian, P.S., Vignal-Clermont, C., Wang, A.G., Donahue, S.P., Leroy, B.P., et al. (2023). Indirect Comparison of Lenadogene Nolparvovec Gene Therapy Versus Natural History in Patients with Leber Hereditary Optic Neuropathy Carrying the m.11778G>A MT-ND4 Mutation. Ophthalmol. Ther. *12*, 401–429. https://doi.org/10.1007/s40123-022-00611-x.
21. Lake, N.J., Compton, A.G., Rahman, S., and Thorburn, D.R. (2016). Leigh syndrome: One disorder, more than 75 monogenic causes. Ann. Neurol. *79*, 190–203. https://doi.org/10.1002/ana.24551.
22. Naeem, M.M., Maheshan, R., Costford, S.R., Wahedi, A., Trajkovski, M., Plavec, J., Yatsunyk, L.A., Ciesielski, G.L., Kaufman, B.A., and Sondheimer, N. (2019). G-quadruplex-mediated reduction of a pathogenic mitochondrial heteroplasmy. Hum. Mol. Genet. *28*, 3163–3174. https://doi.org/10.1093/hmg/ddz153.
23. Hashimoto, M., Bacman, S.R., Peralta, S., Falk, M.J., Chomyn, A., Chan, D.C., Williams, S.L., and Moraes, C.T. (2015). MitoTALEN: A General Approach to Reduce Mutant mtDNA Loads and Restore Oxidative Phosphorylation Function in Mitochondrial Diseases. Mol. Ther. *23*, 1592–1599. https://doi.org/10.1038/mt.2015.126.
24. Yahata, N., Boda, H., and Hata, R. (2021). Elimination of Mutant mtDNA by an Optimized mpTALEN Restores Differentiation Capacities of

CellPress
OPEN ACCESS

Heteroplasmic MELAS-iPSCs. Mol. Ther. Methods Clin. Dev. *20*, 54–68. https://doi.org/10.1016/j.omtm.2020.10.017.

25. Yahata, N., Matsumoto, Y., Omi, M., Yamamoto, N., and Hata, R. (2017). TALEN-mediated shift of mitochondrial DNA heteroplasmy in MELAS-iPSCs with m.13513G>A mutation. Sci. Rep. *7*, 15557. https://doi.org/10.1038/s41598-017-15871-y.

26. Tanaka, M., Borgeld, H.J., Zhang, J., Muramatsu, S.i., Gong, J.S., Yoneda, M., Maruyama, W., Naoi, M., Ibi, T., Sahashi, K., et al. (2002). Gene therapy for mitochondrial disease by delivering restriction endonuclease SmaI into mitochondria. J. Biomed. Sci. *9*, 534–541. https://doi.org/10.1159/000064726.

27. Alexeyev, M.F., Venediktova, N., Pastukh, V., Shokolenko, I., Bonilla, G., and Wilson, G.L. (2008). Selective elimination of mutant mitochondrial genomes as therapeutic strategy for the treatment of NARP and MILS syndromes. Gene Ther. *15*, 516–523. https://doi.org/10.1038/gt.2008.11.

28. Minczuk, M., Papworth, M.A., Miller, J.C., Murphy, M.P., and Klug, A. (2008). Development of a single-chain, quasi-dimeric zinc-finger nuclease for the selective degradation of mutated human mitochondrial DNA. Nucleic Acids Res. *36*, 3926–3938. https://doi.org/10.1093/nar/gkn313.

29. Gammage, P.A., Rorbach, J., Vincent, A.I., Rebar, E.J., and Minczuk, M. (2014). Mitochondrially targeted ZFNs for selective degradation of pathogenic mitochondrial genomes bearing large-scale deletions or point mutations. EMBO Mol. Med. *6*, 458–466. https://doi.org/10.1002/emmm.201303672.

30. Gammage, P.A., Gaude, E., Van Haute, L., Rebelo-Guiomar, P., Jackson, C.B., Rorbach, J., Pekalski, M.L., Robinson, A.J., Charpentier, M., Concordet, J.P., et al. (2016). Near-complete elimination of mutant mtDNA by iterative or dynamic dose-controlled treatment with mtZFNs. Nucleic Acids Res. *44*, 7804–7816. https://doi.org/10.1093/nar/gkw676.

31. Gaude, E., Schmidt, C., Gammage, P.A., Dugourd, A., Blacker, T., Chew, S.P., Saez-Rodriguez, J., O'Neill, J.S., Szabadkai, G., Minczuk, M., and Frezza, C. (2018). NADH Shuttling Couples Cytosolic Reductive Carboxylation of Glutamine with Glycolysis in Cells with Mitochondrial Dysfunction. Mol. Cell *69*, 581–593.e7. https://doi.org/10.1016/j.molcel.2018.01.034.

32. Bruno, C., Martinuzzi, A., Tang, Y., Andreu, A.L., Pallotti, F., Bonilla, E., Shanske, S., Fu, J., Sue, C.M., Angelini, C., et al. (1999). A stop-codon mutation in the human mtDNA cytochrome c oxidase I gene disrupts the functional structure of complex IV. Am. J. Hum. Genet. *65*, 611–620. https://doi.org/10.1086/302546.

33. Yang, Q., Liu, P., Anderson, N.S., Shpilka, T., Du, Y., Naresh, N.U., Li, R., Zhu, L.J., Luk, K., Lavelle, J., et al. (2022). LONP-1 and ATFS-1 sustain deleterious heteroplasmy by promoting mtDNA replication in dysfunctional mitochondria. Nat. Cell Biol. *24*, 181–193. https://doi.org/10.1038/s41556-021-00840-5.

34. Pearson, H.A., Lobel, J.S., Kocoshis, S.A., Naiman, J.L., Windmiller, J., Lammi, A.T., Hoffman, R., and Marsh, J.C. (1979). A new syndrome of refractory sideroblastic anemia with vacuolization of marrow precursors and exocrine pancreatic dysfunction. J. Pediatr. *95*, 976–984. https://doi.org/10.1016/s0022-3476(79)80286-3.

35. Yoshimi, A., Ishikawa, K., Niemeyer, C., and Grünert, S.C. (2022). Pearson syndrome: a multisystem mitochondrial disease with bone marrow failure. Orphanet J. Rare Dis. *17*, 379. https://doi.org/10.1186/s13023-022-02538-9.

36. Bacman, S.R., Williams, S.L., Pinto, M., Peralta, S., and Moraes, C.T. (2013). Specific elimination of mutant mitochondrial genomes in patient-derived cells by mitoTALENs. Nat. Med. *19*, 1111–1113. https://doi.org/10.1038/nm.3261.

37. Pavlakis, S.G., Phillips, P.C., DiMauro, S., De Vivo, D.C., and Rowland, L.P. (1984). Mitochondrial myopathy, encephalopathy, lactic acidosis, and strokelike episodes: a distinctive clinical syndrome. Ann. Neurol. *16*, 481–488. https://doi.org/10.1002/ana.410160409.

38. El-Hattab, A.W., Almannai, M., and Scaglia, F. (2001). MELAS, M.P. Adam, S. Bick, G.M. Mirzaa, R.A. Pagon, S.E. Wallace, and A. Amemiya, eds. (Seattle: GeneReviews. University of Washington), pp. 1993–2026.

39. Shoop, W.K., Lape, J., Trum, M., Powell, A., Sevigny, E., Mischler, A., Bacman, S.R., Fontanesi, F., Smith, J., Jantz, D., et al. (2023). Efficient elimination of MELAS-associated m.3243G mutant mitochondrial DNA by an engineered mitoARCUS nuclease. Nat. Metab. *5*, 2169–2183. https://doi.org/10.1038/s42255-023-00932-6.

40. Yang, Y., Wu, H., Kang, X., Liang, Y., Lan, T., Li, T., Tan, T., Peng, J., Zhang, Q., An, G., et al. (2018). Targeted elimination of mutant mitochondrial DNA in MELAS-iPSCs by mitoTALENs. Protein Cell *9*, 283–297. https://doi.org/10.1007/s13238-017-0499-y.

41. Yahata, N., Goto, Y.I., and Hata, R. (2025). Optimization of mtDNA-targeted platinum TALENs for bi-directionally modifying heteroplasmy levels in patient-derived m.3243A>G-iPSCs. Mol. Ther. Nucleic Acids *36*, 102521. https://doi.org/10.1016/j.omtn.2025.102521.

42. Chung, C.Y., Singh, K., Kotiadis, V.N., Valdebenito, G.E., Ahn, J.H., Topley, E., Tan, J., Andrews, W.D., Bilanges, B., Pitceathly, R.D.S., et al. (2021). Constitutive activation of the PI3K-Akt-mTORC1 pathway sustains the m.3243 A > G mtDNA mutation. Nat. Commun. *12*, 6409. https://doi.org/10.1038/s41467-021-26746-2.

43. Chung, C.Y., Singh, K., Sheshadri, P., Valdebenito, G.E., Chacko, A.R., Costa Besada, M.A., Liang, X.F., Kabir, L., Pitceathly, R.D.S., Szabadkai, G., and Duchen, M.R. (2025). Inhibition of the PI3K-AKT-MTORC1 axis reduces the burden of the m.3243A>G mtDNA mutation by promoting mitophagy and improving mitochondrial function. Autophagy *21*, 881–896. https://doi.org/10.1080/15548627.2024.2437908.

44. Pantic, B., Ives, D., Mennuni, M., Perez-Rodriguez, D., Fernandez-Pelayo, U., Lopez de Arbina, A., Muñoz-Oreja, M., Villar-Fernandez, M., Dang, T.M.J., Vergani, L., et al. (2021). 2-Deoxy-D-glucose couples mitochondrial DNA replication with mitochondrial fitness and promotes the selection of wild-type over mutant mitochondrial DNA. Nat. Commun. *12*, 6997. https://doi.org/10.1038/s41467-021-26829-0.

45. Kauppila, J.H.K., Baines, H.L., Bratic, A., Simard, M.L., Freyer, C., Mourier, A., Stamp, C., Filograna, R., Larsson, N.G., Greaves, L.C., and Stewart, J.B. (2016). A Phenotype-Driven Approach to Generate Mouse Models with Pathogenic mtDNA Mutations Causing Mitochondrial Disease. Cell Rep. *16*, 2980–2990. https://doi.org/10.1016/j.celrep.2016.08.037.

46. Nash, P.A., Turner, K.M., Powell, C.A., Van Haute, L., Silva-Pinheiro, P., Bubeck, F., Wiedtke, E., Marques, E., Ryan, D.G., Grimm, D., et al. (2025). Clinically translatable mitochondrial gene therapy in muscle using tandem mtZFN architecture. EMBO Mol. Med. *17*, 1222–1237. https://doi.org/10.1038/s44321-025-00231-5.

47. Gammage, P.A., Viscomi, C., Simard, M.L., Costa, A.S.H., Gaude, E., Powell, C.A., Van Haute, L., McCann, B.J., Rebelo-Guiomar, P., Cerutti, R., et al. (2018). Genome editing in mitochondria corrects a pathogenic mtDNA mutation in vivo. Nat. Med. *24*, 1691–1695. https://doi.org/10.1038/s41591-018-0165-9.

48. Bacman, S.R., Kauppila, J.H.K., Pereira, C.V., Nissanka, N., Miranda, M., Pinto, M., Williams, S.L., Larsson, N.G., Stewart, J.B., and Moraes, C.T. (2018). MitoTALEN reduces mutant mtDNA load and restores tRNA(Ala) levels in a mouse model of heteroplasmic mtDNA mutation. Nat. Med. *24*, 1696–1700. https://doi.org/10.1038/s41591-018-0166-8.

49. Zekonyte, U., Bacman, S.R., Smith, J., Shoop, W., Pereira, C.V., Tomberlin, G., Stewart, J., Jantz, D., and Moraes, C.T. (2021). Mitochondrial targeted meganuclease as a platform to eliminate mutant mtDNA in vivo. Nat. Commun. *12*, 3210. https://doi.org/10.1038/s41467-021-23561-7.

50. Barrera-Paez, J.D., Bacman, S.R., Balla, T., Van Booven, D., Gannamedi, D.P., Stewart, J.B., Mok, B., Liu, D.R., Lombard, D.B., Griswold, A.J., et al. (2025). Correcting a pathogenic mitochondrial DNA mutation by base editing in mice. Sci. Transl. Med. *17*, eadr0792. https://doi.org/10.1126/scitranslmed.adr0792.

51. Sengupta, A., Akhtar, M.M., Akhtar, A.M., Lorenzini, M., Slater, T., Syrris, P., Savvatis, K., Bakalakos, A., and Elliott, P.M. (2025). Cardiomyopathy caused by mitochondrial DNA 4300A > G variant. Eur. Heart J. *46*, 3118–3121. https://doi.org/10.1093/eurheartj/ehaf319.

52. Chen, X., Chen, M., Zhu, Y., Sun, H., Wang, Y., Xie, Y., Ji, L., Wang, C., Hu, Z., Guo, X., et al. (2023). Correction of a homoplasmic mitochondrial tRNA mutation in patient-derived iPSCs via a mitochondrial base editor. Commun. Biol. *6*, 1116. https://doi.org/10.1038/s42003-023-05500-y.

53. Chen, B.S., and Yu-Wai-Man, P. (2022). From Bench to Bedside-Delivering Gene Therapy for Leber Hereditary Optic Neuropathy. Cold Spring Harb. Perspect. Med. *12*, a041282. https://doi.org/10.1101/cshperspect.a041282.

54. Yu, H., Koilkonda, R.D., Chou, T.H., Porciatti, V., Ozdemir, S.S., Chiodo, V., Boye, S.L., Boye, S.E., Hauswirth, W.W., Lewin, A.S., and Guy, J. (2012). Gene delivery to mitochondria by targeting modified adenoassociated virus suppresses Leber's hereditary optic neuropathy in a mouse model. Proc. Natl. Acad. Sci. USA *109*, E1238–E1247. https://doi.org/10.1073/pnas.1119577109.

55. Yang, S., Ma, S.Q., Wan, X., He, H., Pei, H., Zhao, M.J., Chen, C., Wang, D.W., Dong, X.Y., Yuan, J.J., and Li, B. (2016). Long-term outcomes of gene therapy for the treatment of Leber's hereditary optic neuropathy. EBioMedicine *10*, 258–268. https://doi.org/10.1016/j.ebiom.2016.07.002.

56. Yu-Wai-Man, P., Newman, N.J., Carelli, V., Moster, M.L., Biousse, V., Sadun, A.A., Klopstock, T., Vignal-Clermont, C., Sergott, R.C., Rudolph, G., et al. (2020). Bilateral visual improvement with unilateral gene therapy injection for Leber hereditary optic neuropathy. Sci. Transl. Med. *12*, eaaz7423. https://doi.org/10.1126/scitranslmed.aaz7423.

57. Newman, N.J., Yu-Wai-Man, P., Carelli, V., Biousse, V., Moster, M.L., Vignal-Clermont, C., Sergott, R.C., Klopstock, T., Sadun, A.A., Girmens, J.F., et al. (2021). Intravitreal Gene Therapy vs. Natural History in Patients With Leber Hereditary Optic Neuropathy Carrying the m.11778G>A ND4 Mutation: Systematic Review and Indirect Comparison. Front. Neurol. *12*, 662838. https://doi.org/10.3389/fneur.2021.662838.

58. Newman, N.J., Yu-Wai-Man, P., Carelli, V., Moster, M.L., Biousse, V., Vignal-Clermont, C., Sergott, R.C., Klopstock, T., Sadun, A.A., Barboni, P., et al. (2021). Efficacy and Safety of Intravitreal Gene Therapy for Leber Hereditary Optic Neuropathy Treated within 6 Months of Disease Onset. Ophthalmology *128*, 649–660. https://doi.org/10.1016/j.ophtha.2020.12.012.

59. Biousse, V., Newman, N.J., Yu-Wai-Man, P., Carelli, V., Moster, M.L., Vignal-Clermont, C., Klopstock, T., Sadun, A.A., Sergott, R.C., Hage, R., et al. (2021). Long-Term Follow-Up After Unilateral Intravitreal Gene Therapy for Leber Hereditary Optic Neuropathy: The RESTORE Study. J. Neuro Ophthalmol. *41*, 309–315. https://doi.org/10.1097/WNO.0000000000001367.

60. Yu-Wai-Man, P., Newman, N., Subramanian, P.S., Moster, M., Wang, A.G., Donahue, S., Leroy, B., Carelli, V., Biousse, V., Vignal-Clermont, C., et al. (2022). The phase III REFLECT trial: efficacy of bilateral gene therapy for Leber hereditary optic neuropathy (LHON) is maintained 2 years post administration. Invest. Ophth. Vis. Sci. *63*, 434.

61. Newman, N.J., Yu-Wai-Man, P., Subramanian, P.S., Moster, M.L., Wang, A.G., Donahue, S.P., Leroy, B.P., Carelli, V., Biousse, V., Vignal-Clermont, C., et al. (2023). Randomized trial of bilateral gene therapy injection for m.11778G>A MT-ND4 Leber optic neuropathy. Brain *146*, 1328–1341. https://doi.org/10.1093/brain/awac421.

62. Yi, Z., Zhang, X., Tang, W., Yu, Y., Wei, X., Zhang, X., and Wei, W. (2024). Strand-selective base editing of human mitochondrial DNA using mitoBEs. Nat. Biotechnol. *42*, 498–509. https://doi.org/10.1038/s41587-023-01791-y.

63. Kim, S., Kim, J., Cha, S., Ju, S., Lim, C.J., Hong, S., Bae, J., Oh, Y., Jung, S., Kim, S.P., et al. (2025). In vivo mitochondrial base editing restores genotype and visual function in a mouse model of LHON. Nat. Commun. *16*, 10923. https://doi.org/10.1038/s41467-025-66600-3.

64. Dai, Y., Zheng, K., Clark, J., Swerdlow, R.H., Pulst, S.M., Sutton, J.P., Shinobu, L.A., and Simon, D.K. (2014). Rapamycin drives selection against a pathogenic heteroplasmic mitochondrial DNA mutation. Hum. Mol. Genet. *23*, 637–647. https://doi.org/10.1093/hmg/ddt450.

65. Reddy, P., Ocampo, A., Suzuki, K., Luo, J., Bacman, S.R., Williams, S.L., Sugawara, A., Okamura, D., Tsunekawa, Y., Wu, J., et al. (2015). Selective elimination of mitochondrial mutations in the germline by genome editing. Cell *161*, 459–469. https://doi.org/10.1016/j.cell.2015.03.051.

66. Wang, J., Ji, Y., Ai, C., Chen, J.R., Gan, D., Zhang, J., Mo, J.Q., and Guan, M.X. (2023). Optimized allotopic expression of mitochondrial ND6 transgene restored complex I and apoptosis deficiencies caused by LHON-linked ND6 14484T > C mutation. J. Biomed. Sci. *30*, 63. https://doi.org/10.1186/s12929-023-00951-1.

67. Stendel, C., Neuhofer, C., Floride, E., Yuqing, S., Ganetzky, R.D., Park, J., Freisinger, P., Kornblum, C., Kleinle, S., Schöls, L., et al. (2020). Delineating MT-ATP6-associated disease: From isolated neuropathy to early onset neurodegeneration. Neurol. Genet. *6*, e393. https://doi.org/10.1212/NXG.0000000000000393.

68. Finsterer, J. (2023). Neuropathy, Ataxia, and Retinitis Pigmentosa Syndrome. J. Clin. Neuromuscul. Dis. *24*, 140–146. https://doi.org/10.1097/CND.0000000000000422.

69. Goldstein, A., and Falk, M.J. (2003). Single Large-Scale Mitochondrial DNA Deletion Syndromes, M.P. Adam, S. Bick, G.M. Mirzaa, R.A. Pagon, S.E. Wallace, and A. Amemiya, eds. (Seattle: GeneReviews University of Washington), pp. 1993–2026.

70. Goldstein, A., and Falk, M.J. (2003). Single Large-Scale Mitochondrial DNA Deletion Syndromes, M.P. Adam, S. Bick, G.M. Mirzaa, R.A. Pagon, S.E. Wallace, and A. Amemiya, eds. (Seattle: GeneReviews University of Washington), pp. 1993–2026.

71. Pereira, C.V., Bacman, S.R., Arguello, T., Zekonyte, U., Williams, S.L., Edgell, D.R., and Moraes, C.T. (2018). mitoTev-TALE: a monomeric DNA editing enzyme to reduce mutant mitochondrial DNA levels. EMBO Mol. Med. *10*, e8084. https://doi.org/10.15252/emmm.201708084.

72. Viering, D., Schlingmann, K.P., Hureaux, M., Nijenhuis, T., Mallett, A., Chan, M.M.Y., van Beek, A., van Eerde, A.M., Coulibaly, J.M., Vallet, M., et al. (2022). Gitelman-Like Syndrome Caused by Pathogenic Variants in mtDNA. J. Am. Soc. Nephrol. *33*, 305–325. https://doi.org/10.1681/ASN.2021050596.

73. Silva-Pinheiro, P., and Minczuk, M. (2022). The potential of mitochondrial genome engineering. Nat. Rev. Genet. *23*, 199–214. https://doi.org/10.1038/s41576-021-00432-x.

74. Zorova, L.D., Popkov, V.A., Plotnikov, E.Y., Silachev, D.N., Pevzner, I.B., Jankauskas, S.S., Babenko, V.A., Zorov, S.D., Balakireva, A.V., Juhaszova, M., et al. (2018). Mitochondrial membrane potential. Anal. Biochem. *552*, 50–59. https://doi.org/10.1016/j.ab.2017.07.009.

75. Stewart, J.B., and Chinnery, P.F. (2021). Extreme heterogeneity of human mitochondrial DNA from organelles to populations. Nat. Rev. Genet. *22*, 106–118. https://doi.org/10.1038/s41576-020-00284-x.

76. Smith, K.K., Moreira, J.D., Wilson, C.R., Padera, J.O., Lamason, A.N., Xue, L., Gopal, D.M., Flynn, D.B., and Fetterman, J.L. (2024). A systematic review on the biochemical threshold of mitochondrial genetic variants. Genome Res. *34*, 341–365. https://doi.org/10.1101/gr.278200.123.

77. Panades-de Oliveira, L., Montoya, J., Emperador, S., Ruiz-Pesini, E., Jerico, I., Arenas, J., Hernandez-Lain, A., Blazquez, A., Martin, M.A., and Dominguez-Gonzalez, C. (2020). A novel mutation in the mitochondrial MT-ND5 gene in a family with MELAS. The relevance of genetic analysis on targeted tissues. Mitochondrion *50*, 14–18. https://doi.org/10.1016/j.mito.2019.10.001.

78. Sacconi, S., Salviati, L., Nishigaki, Y., Walker, W.F., Hernandez-Rosa, E., Trevisson, E., Delplace, S., Desnuelle, C., Shanske, S., Hirano, M., et al. (2008). A functionally dominant mitochondrial DNA mutation. Hum. Mol. Genet. *17*, 1814–1820. https://doi.org/10.1093/hmg/ddn073.

CellPress
OPEN ACCESS

79. Jeppesen, T.D., Duno, M., and Vissing, J. (2020). Mutation Load of Single, Large-Scale Deletions of mtDNA in Mitotic and Postmitotic Tissues. Front. Genet. *11*, 547638. https://doi.org/10.3389/fgene.2020.547638.

80. Broomfield, A., Sweeney, M.G., Woodward, C.E., Fratter, C., Morris, A.M., Leonard, J.V., Abulhoul, L., Grunewald, S., Clayton, P.T., Hanna, M.G., et al. (2015). Paediatric single mitochondrial DNA deletion disorders: an overlapping spectrum of disease. J. Inherit. Metab. Dis. *38*, 445–457. https://doi.org/10.1007/s10545-014-9778-4.

81. Chiaratti, M.R., and Chinnery, P.F. (2022). Modulating mitochondrial DNA mutations: factors shaping heteroplasmy in the germ line and somatic cells. Pharmacol. Res. *185*, 106466. https://doi.org/10.1016/j.phrs.2022.106466.

82. Grady, J.P., Pickett, S.J., Ng, Y.S., Alston, C.L., Blakely, E.L., Hardy, S.A., Feeney, C.L., Bright, A.A., Schaefer, A.M., Gorman, G.S., et al. (2018). mtDNA heteroplasmy level and copy number indicate disease burden in m.3243A>G mitochondrial disease. EMBO Mol. Med. *10*, e8262. https://doi.org/10.15252/emmm.201708262.

83. Hage, R., and Vignal-Clermont, C. (2021). Leber Hereditary Optic Neuropathy: Review of Treatment and Management. Front. Neurol. *12*, 651639. https://doi.org/10.3389/fneur.2021.651639.

84. Endo, T., and Wiedemann, N. (2025). Molecular machineries and pathways of mitochondrial protein transport. Nat. Rev. Mol. Cell Biol. *26*, 848–867. https://doi.org/10.1038/s41580-025-00865-w.

85. Magalhães, P.J., Andreu, A.L., and Schon, E.A. (1998). Evidence for the presence of 5S rRNA in mammalian mitochondria. Mol. Biol. Cell *9*, 2375–2382. https://doi.org/10.1091/mbc.9.9.2375.

86. Schneider, A. (2011). Mitochondrial tRNA import and its consequences for mitochondrial translation. Annu. Rev. Biochem. *80*, 1033–1053. https://doi.org/10.1146/annurev-biochem-060109-092838.

87. Cruz-Zaragoza, L.D., Dahal, D., Koschel, M., Boshnakovska, A., Zheenbekova, A., Yilmaz, M., Morgenstern, M., Dohrke, J.N., Bender, J., Valpadashi, A., et al. (2025). Silencing mitochondrial gene expression in living cells. Science *389*, eadr3498. https://doi.org/10.1126/science.adr3498.

88. Gammage, P.A., Moraes, C.T., and Minczuk, M. (2018). Mitochondrial Genome Engineering: The Revolution May Not Be CRISPR-Ized. Trends Genet. *34*, 101–110. https://doi.org/10.1016/j.tig.2017.11.001.

89. Yu-Wai-Man, P., Griffiths, P.G., and Chinnery, P.F. (2011). Mitochondrial optic neuropathies - disease mechanisms and therapeutic strategies. Prog. Retin. Eye Res. *30*, 81–114. https://doi.org/10.1016/j.preteyeres.2010.11.002.

90. Artika, I.M. (2020). Allotopic expression of mitochondrial genes: Basic strategy and progress. Genes Dis. *7*, 578–584. https://doi.org/10.1016/j.gendis.2019.08.001.

91. Lewis, C.J., Dixit, B., Batiuk, E., Hall, C.J., O'Connor, M.S., and Boominathan, A. (2020). Codon optimization is an essential parameter for the efficient allotopic expression of mtDNA genes. Redox Biol. *30*, 101429. https://doi.org/10.1016/j.redox.2020.101429.

92. Nieto-Panqueva, F., Rubalcava-Gracia, D., Hamel, P.P., and González-Halphen, D. (2023). The constraints of allotopic expression. Mitochondrion *73*, 30–50. https://doi.org/10.1016/j.mito.2023.09.004.

93. Moretton, A., Morel, F., Macao, B., Lachaume, P., Ishak, L., Lefebvre, M., Garreau-Balandier, I., Vernet, P., Falkenberg, M., and Farge, G. (2017). Selective mitochondrial DNA degradation following double-strand breaks. PLoS One *12*, e0176795. https://doi.org/10.1371/journal.pone.0176795.

94. Peeva, V., Blei, D., Trombly, G., Corsi, S., Szukszto, M.J., Rebelo-Guiomar, P., Gammage, P.A., Kudin, A.P., Becker, C., Altmüller, J., et al. (2018). Linear mitochondrial DNA is rapidly degraded by components of the replication machinery. Nat. Commun. *9*, 1727. https://doi.org/10.1038/s41467-018-04131-w.

95. Nissanka, N., Bacman, S.R., Plastini, M.J., and Moraes, C.T. (2018). The mitochondrial DNA polymerase gamma degrades linear DNA fragments precluding the formation of deletions. Nat. Commun. *9*, 2491. https://doi.org/10.1038/s41467-018-04895-1.

96. Bayona-Bafaluy, M.P., Blits, B., Battersby, B.J., Shoubridge, E.A., and Moraes, C.T. (2005). Rapid directional shift of mitochondrial DNA heteroplasmy in animal tissues by a mitochondrially targeted restriction endonuclease. Proc. Natl. Acad. Sci. USA *102*, 14392–14397. https://doi.org/10.1073/pnas.0502896102.

97. Bacman, S.R., Williams, S.L., Duan, D., and Moraes, C.T. (2012). Manipulation of mtDNA heteroplasmy in all striated muscles of newborn mice by AAV9-mediated delivery of a mitochondria-targeted restriction endonuclease. Gene Ther. *19*, 1101–1106. https://doi.org/10.1038/gt.2011.196.

98. Miller, J.C., Patil, D.P., Xia, D.F., Paine, C.B., Fauser, F., Richards, H.W., Shivak, D.A., Bendaña, Y.R., Hinkley, S.J., Scarlott, N.A., et al. (2019). Enhancing gene editing specificity by attenuating DNA cleavage kinetics. Nat. Biotechnol. *37*, 945–952. https://doi.org/10.1038/s41587-019-0186-z.

99. Paschon, D.E., Lussier, S., Wangzor, T., Xia, D.F., Li, P.W., Hinkley, S.J., Scarlott, N.A., Lam, S.C., Waite, A.J., Truong, L.N., et al. (2019). Diversifying the structure of zinc finger nucleases for high-precision genome editing. Nat. Commun. *10*, 1133. https://doi.org/10.1038/s41467-019-08867-x.

100. Katayama, S., Watanabe, M., Kato, Y., Nomura, W., and Yamamoto, T. (2024). Engineering of Zinc Finger Nucleases Through Structural Modeling Improves Genome Editing Efficiency in Cells. Adv. Sci. *11*, e2310255. https://doi.org/10.1002/advs.202310255.

101. Becker, S., and Boch, J. (2021). TALE and TALEN genome editing technologies. Gene and Genome Editing *2*, 100007. https://doi.org/10.1016/j.ggedit.2021.100007.

102. Xie, L., Feng, H., Li, Z., Li, D., Yang, X., Yuan, T., Yan, N., He, C., Zheng, J., Zuo, Z., et al. (2024). Undetectable off-target effects induced by FokI catalytic domain in mouse embryos. Genome Biol. *25*, 51. https://doi.org/10.1186/s13059-024-03188-9.

103. Hou, X., Zaks, T., Langer, R., and Dong, Y. (2021). Lipid nanoparticles for mRNA delivery. Nat. Rev. Mater. *6*, 1078–1094. https://doi.org/10.1038/s41578-021-00358-0.

104. Yamada, Y., Ishizuka, S., Arai, M., Maruyama, M., and Harashima, H. (2022). Recent advances in delivering RNA-based therapeutics to mitochondria. Expert Opin. Biol. Ther. *22*, 1209–1219. https://doi.org/10.1080/14712598.2022.2070427.

105. Kawamura, E., Maruyama, M., Abe, J., Sudo, A., Takeda, A., Takada, S., Yokota, T., Kinugawa, S., Harashima, H., and Yamada, Y. (2020). Validation of Gene Therapy for Mutant Mitochondria by Delivering Mitochondrial RNA Using a MITO-Porter. Mol. Ther. Nucleic Acids *20*, 687–698. https://doi.org/10.1016/j.omtn.2020.04.004.

106. Mok, B.Y., de Moraes, M.H., Zeng, J., Bosch, D.E., Kotrys, A.V., Raguram, A., Hsu, F., Radey, M.C., Peterson, S.B., Mootha, V.K., et al. (2020). A bacterial cytidine deaminase toxin enables CRISPR-free mitochondrial base editing. Nature *583*, 631–637. https://doi.org/10.1038/s41586-020-2477-4.

107. Lim, K., Cho, S.I., and Kim, J.S. (2022). Nuclear and mitochondrial DNA editing in human cells with zinc finger deaminases. Nat. Commun. *13*, 366. https://doi.org/10.1038/s41467-022-27962-0.

108. Cho, S.I., Lee, S., Mok, Y.G., Lim, K., Lee, J., Lee, J.M., Chung, E., and Kim, J.S. (2022). Targeted A-to-G base editing in human mitochondrial DNA with programmable deaminases. Cell *185*, 1764–1776.e12. https://doi.org/10.1016/j.cell.2022.03.039.

109. Hu, J., Sun, Y., Li, B., Liu, Z., Wang, Z., Gao, Q., Guo, M., Liu, G., Zhao, K.T., and Gao, C. (2024). Strand-preferred base editing of organellar and nuclear genomes using CyDENT. Nat. Biotechnol. *42*, 936–945. https://doi.org/10.1038/s41587-023-01910-9.

110. Chen, X., Liang, D., Guo, J., Zhang, J., Sun, H., Zhang, X., Jin, J., Dai, Y., Bao, Q., Qian, X., et al. (2022). DdCBE-mediated mitochondrial base

editing in human 3PN embryos. Cell Discov. *8*, 8. https://doi.org/10.1038/s41421-021-00358-y.

111. Lee, H., Lee, S., Baek, G., Kim, A., Kang, B.C., Seo, H., and Kim, J.S. (2021). Mitochondrial DNA editing in mice with DddA-TALE fusion deaminases. Nat. Commun. *12*, 1190. https://doi.org/10.1038/s41467-021-21464-1.

112. Silva-Pinheiro, P., Mutti, C.D., Van Haute, L., Powell, C.A., Nash, P.A., Turner, K., and Minczuk, M. (2023). A library of base editors for the precise ablation of all protein-coding genes in the mouse mitochondrial genome. Nat. Biomed. Eng. *7*, 692–703. https://doi.org/10.1038/s41551-022-00968-1.

113. Guo, J., Yu, W., Li, M., Chen, H., Liu, J., Xue, X., Lin, J., Huang, S., Shu, W., Huang, X., et al. (2023). A DddA ortholog-based and transactivator-assisted nuclear and mitochondrial cytosine base editors with expanded target compatibility. Mol. Cell *83*, 1710–1724.e7. https://doi.org/10.1016/j.molcel.2023.04.012.

114. Silva-Pinheiro, P., Nash, P.A., Van Haute, L., Mutti, C.D., Turner, K., and Minczuk, M. (2022). In vivo mitochondrial base editing via adeno-associated viral delivery to mouse post-mitotic tissue. Nat. Commun. *13*, 750. https://doi.org/10.1038/s41467-022-28358-w.

115. Guo, J., Zhang, X., Chen, X., Sun, H., Dai, Y., Wang, J., Qian, X., Tan, L., Lou, X., and Shen, B. (2021). Precision modeling of mitochondrial diseases in zebrafish via DdCBE-mediated mtDNA base editing. Cell Discov. *7*, 78. https://doi.org/10.1038/s41421-021-00307-9.

116. Sabharwal, A., Kar, B., Restrepo-Castillo, S., Holmberg, S.R., Mathew, N.D., Kendall, B.L., Cotter, R.P., WareJoncas, Z., Seiler, C., Nakamaru-Ogiso, E., et al. (2021). The FusX TALE Base Editor (FusXTBE) for Rapid Mitochondrial DNA Programming of Human Cells In Vitro and Zebrafish Disease Models In Vivo. CRISPR J. *4*, 799–821. https://doi.org/10.1089/crispr.2021.0061.

117. Bacman, S.R., and Moraes, C.T. (2020). Mitochondrial DNA Base Editing: Good Editing Things Still Come in Small Packages. Mol. Cell *79*, 708–709. https://doi.org/10.1016/j.molcel.2020.08.009.

118. Lei, Z., Meng, H., Liu, L., Zhao, H., Rao, X., Yan, Y., Wu, H., Liu, M., He, A., and Yi, C. (2022). Mitochondrial base editor induces substantial nuclear off-target mutations. Nature *606*, 804–811. https://doi.org/10.1038/s41586-022-04836-5.

119. Mok, Y.G., Lee, J.M., Chung, E., Lee, J., Lim, K., Cho, S.I., and Kim, J.S. (2022). Base editing in human cells with monomeric DddA-TALE fusion deaminases. Nat. Commun. *13*, 4038. https://doi.org/10.1038/s41467-022-31745-y.

120. Castillo, S.R., Simone, B.W., Clark, K.J., Devaux, P., and Ekker, S.C. (2024). Unconstrained Precision Mitochondrial Genome Editing with alphaDdCBEs. Hum. Gene Ther. *35*, 798–813. https://doi.org/10.1089/hum.2024.073.

121. Mi, L., Shi, M., Li, Y.X., Xie, G., Rao, X., Wu, D., Cheng, A., Niu, M., Xu, F., Yu, Y., et al. (2023). DddA homolog search and engineering expand sequence compatibility of mitochondrial base editing. Nat. Commun. *14*, 874. https://doi.org/10.1038/s41467-023-36600-2.

122. Liu, J., Chen, J., Huang, S., Guo, J., Li, X., Yan, Y., Chen, R., Li, G., Liu, M., Wei, J., et al. (2025). An engineered mitoCBE facilitates efficient mitochondrial DNA editing and modified mitochondrial transfer. Mol. Ther. *33*, 3114–3127. https://doi.org/10.1016/j.ymthe.2025.03.051.

123. Kweon, J., Park, S., Jeon, M.Y., Lim, K., Jang, G., Jang, A.H., Lee, M., Seok, C., Lee, C., Park, S., et al. (2025). High-efficiency base editing for nuclear and mitochondrial DNA with an optimized DYW-like deaminase. Mol. Ther. *33*, 5611–5623. https://doi.org/10.1016/j.ymthe.2025.08.007.

124. Mok, B.Y., Kotrys, A.V., Raguram, A., Huang, T.P., Mootha, V.K., and Liu, D.R. (2022). CRISPR-free base editors with enhanced activity and expanded targeting scope in mitochondrial and nuclear DNA. Nat. Biotechnol. *40*, 1378–1387. https://doi.org/10.1038/s41587-022-01256-8.

125. Lee, S., Lee, H., Baek, G., and Kim, J.S. (2023). Precision mitochondrial DNA editing with high-fidelity DddA-derived base editors. Nat. Biotechnol. *41*, 378–386. https://doi.org/10.1038/s41587-022-01486-w.

126. Willis, J.C.W., Silva-Pinheiro, P., Widdup, L., Minczuk, M., and Liu, D.R. (2022). Compact zinc finger base editors that edit mitochondrial or nuclear DNA in vitro and in vivo. Nat. Commun. *13*, 7204. https://doi.org/10.1038/s41467-022-34784-7.

127. Fan, Y., Xu, W., Gao, B.Q., Qin, H., Wu, X., Wei, J., Ni, Q., Zhou, L., Xiang, J., Wu, J., et al. (2026). Leveraging base excision repair for efficient adenine base editing of mitochondrial DNA. Nat. Biotechnol. *44*, 277–290. https://doi.org/10.1038/s41587-025-02608-w.

128. Wei, Y., Jin, M., Huang, S., Yao, F., Ren, N., Xu, K., Li, S., Gao, P., Zhou, Y., Chen, Y., et al. (2024). Enhanced C-To-T and A-To-G Base Editing in Mitochondrial DNA with Engineered DdCBE and TALED. Adv. Sci. *11*, e2304113. https://doi.org/10.1002/advs.202304113.

129. Cho, S.I., Lim, K., Hong, S., Lee, J., Kim, A., Lim, C.J., Ryou, S., Lee, J.M., Mok, Y.G., Chung, E., et al. (2024). Engineering TALE-linked deaminases to facilitate precision adenine base editing in mitochondrial DNA. Cell *187*, 95–109.e26. https://doi.org/10.1016/j.cell.2023.11.035.

130. Zhang, X., Zhang, X., Ren, J., Li, J., Wei, X., Yu, Y., Yi, Z., and Wei, W. (2025). Precise modelling of mitochondrial diseases using optimized mitoBEs. Nature *639*, 735–745. https://doi.org/10.1038/s41586-024-08469-8.

131. Chen, L., Hong, M., Luan, C., Yuan, M., Wang, Y., Guo, X., Fang, Y., Huang, H., Dong, X., Gao, H., et al. (2026). Efficient mitochondrial A-to-G base editors for the generation of mitochondrial disease models. Nat. Biotechnol. *44*, 418–429. https://doi.org/10.1038/s41587-025-02685-x.

132. Weissig, V. (2020). Drug Development for the Therapy of Mitochondrial Diseases. Trends Mol. Med. *26*, 40–57. https://doi.org/10.1016/j.molmed.2019.09.002.

133. Spinazzola, A., Perez-Rodriguez, D., Ježek, J., and Holt, I.J. (2024). Mitochondrial DNA competition: starving out the mutant genome. Trends Pharmacol. Sci. *45*, 225–242. https://doi.org/10.1016/j.tips.2024.01.011.

134. Palozzi, J.M., Jeedigunta, S.P., Minenkova, A.V., Monteiro, V.L., Thompson, Z.S., Lieber, T., and Hurd, T.R. (2022). Mitochondrial DNA quality control in the female germline requires a unique programmed mitophagy. Cell Metab. *34*, 1809–1823.e6. https://doi.org/10.1016/j.cmet.2022.10.005.

135. Valenzuela, S., Zhu, X., Macao, B., Stamgren, M., Geukens, C., Charifson, P.S., Kern, G., Hoberg, E., Jenninger, L., Gruszczyk, A.V., et al. (2025). Small molecules restore mutant mitochondrial DNA polymerase activity. Nature *642*, 501–507. https://doi.org/10.1038/s41586-025-08856-9.

136. Tostes, K., Dos Santos, A.C., Alves, L.O., Bechara, L.R.G., Marascalchi, R., Macabelli, C.H., Grejo, M.P., Festuccia, W.T., Gottlieb, R.A., Ferreira, J.C.B., and Chiaratti, M.R. (2022). Autophagy deficiency abolishes liver mitochondrial DNA segregation. Autophagy *18*, 2397–2408. https://doi.org/10.1080/15548627.2022.2038501.

137. Falabella, M., Kolesar, J.E., Wallace, C., de Jesus, D., Sun, L., Taguchi, Y.V., Wang, C., Wang, T., Xiang, I.M., Alder, J.K., et al. (2019). G-quadruplex dynamics contribute to regulation of mitochondrial gene expression. Sci. Rep. *9*, 5605. https://doi.org/10.1038/s41598-019-41464-y.

138. de Zeeuw, D., Akizawa, T., Audhya, P., Bakris, G.L., Chin, M., Christ-Schmidt, H., Goldsberry, A., Houser, M., Krauth, M., Lambers Heerspink, H.J., et al. (2013). Bardoxolone methyl in type 2 diabetes and stage 4 chronic kidney disease. N. Engl. J. Med. *369*, 2492–2503. https://doi.org/10.1056/NEJMoa1306033.

139. Panwar, V., Singh, A., Bhatt, M., Tonk, R.K., Azizov, S., Raza, A.S., Sengupta, S., Kumar, D., and Garg, M. (2023). Multifaceted role of mTOR (mammalian target of rapamycin) signaling pathway in human health and disease. Signal Transduct. Target. Ther. *8*, 375. https://doi.org/10.1038/s41392-023-01608-z.

140. Krop, I.E., Mayer, I.A., Ganju, V., Dickler, M., Johnston, S., Morales, S., Yardley, D.A., Melichar, B., Forero-Torres, A., Lee, S.C., et al. (2016).

CellPress
OPEN ACCESS

Pictilisib for oestrogen receptor-positive, aromatase inhibitor-resistant, advanced or metastatic breast cancer (FERGI): a randomised, double-blind, placebo-controlled, phase 2 trial. Lancet Oncol. *17*, 811–821. https://doi.org/10.1016/S1470-2045(16)00106-6.

141. Soria, J.C., Adjei, A.A., Bahleda, R., Besse, B., Ferte, C., Planchard, D., Zhou, J., Ware, J., Morrissey, K., Shankar, G., et al. (2017). A phase IB dose-escalation study of the safety and pharmacokinetics of pictilisib in combination with either paclitaxel and carboplatin (with or without bevacizumab) or pemetrexed and cisplatin (with or without bevacizumab) in patients with advanced non-small cell lung cancer. Eur. J. Cancer *86*, 186–196. https://doi.org/10.1016/j.ejca.2017.08.027.

142. Shapiro, G.I., LoRusso, P., Kwak, E., Pandya, S., Rudin, C.M., Kurkjian, C., Cleary, J.M., Pilat, M.J., Jones, S., de Crespigny, A., et al. (2020). Phase Ib study of the MEK inhibitor cobimetinib (GDC-0973) in combination with the PI3K inhibitor pictilisib (GDC-0941) in patients with advanced solid tumors. Invest. New Drugs *38*, 419–432. https://doi.org/10.1007/s10637-019-00776-6.

143. Gitschlag, B.L., Tate, A.T., and Patel, M.R. (2020). Nutrient status shapes selfish mitochondrial genome dynamics across different levels of selection. eLife *9*, e56686. https://doi.org/10.7554/eLife.56686.

144. Kotrys, A.V., Durham, T.J., Guo, X.A., Vantaku, V.R., Parangi, S., and Mootha, V.K. (2024). Single-cell analysis reveals context-dependent, cell-level selection of mtDNA. Nature *629*, 458–466. https://doi.org/10.1038/s41586-024-07332-0.

145. Chen, Q., Kirk, K., Shurubor, Y.I., Zhao, D., Arreguin, A.J., Shahi, I., Valsecchi, F., Primiano, G., Calder, E.L., Carelli, V., et al. (2018). Rewiring of Glutamine Metabolism Is a Bioenergetic Adaptation of Human Cells with Mitochondrial DNA Mutations. Cell Metab. *27*, 1007–1025.e5. https://doi.org/10.1016/j.cmet.2018.03.002.

146. Raez, L.E., Papadopoulos, K., Ricart, A.D., Chiorean, E.G., Dipaola, R.S., Stein, M.N., Rocha Lima, C.M., Schlesselman, J.J., Tolba, K., Langmuir, V.K., et al. (2013). A phase I dose-escalation trial of 2-deoxy-D-glucose alone or combined with docetaxel in patients with advanced solid tumors. Cancer Chemother. Pharmacol. *71*, 523–530. https://doi.org/10.1007/s00280-012-2045-1.

147. Bischof, T., Gsoellpointner, M., Miljevic, K.D.K., Moser, M.M., Dizdarevic, A.M., Gualdoni, G.A., Gorki, A.D., Nicolodi, C., Chou, S., Radivojev, S., et al. (2025). Safety, tolerability, and pharmacokinetics of intranasal 2-deoxy-D-glucose in normal healthy volunteers: A randomized, double-blind, placebo-controlled, single and multiple ascending dose phase 1 study. Eur. J. Pharm. Sci. *209*, 107069. https://doi.org/10.1016/j.ejps.2025.107069.

148. Bhatt, A.N., Shenoy, S., Munjal, S., Chinnadurai, V., Agarwal, A., Vinoth Kumar, A., Shanavas, A., Kanwar, R., and Chandna, S. (2022). 2-deoxy-D-glucose as an adjunct to standard of care in the medical management of COVID-19: a proof-of-concept and dose-ranging randomised phase II clinical trial. BMC Infect. Dis. *22*, 669. https://doi.org/10.1186/s12879-022-07642-6.

149. Zhou, L., Bao, F., Zheng, J., Ding, Y., Xiao, J., Zhang, J., Qin, Y., Yang, L., Wu, Y., Meng, Q., et al. (2025). Glucose restriction induces degeneration of neurons with mitochondrial DNA depletion by altering ER-mitochondria calcium transfer. Mol. Psychiatry *30*, 4749–4763. https://doi.org/10.1038/s41380-025-03069-y.

150. Castelluccio, N., Spath, K., Li, D., De Coo, I.F.M., Butterworth, L., Wells, D., Mertes, H., Poulton, J., and Heindryckx, B. (2025). Genetic and reproductive strategies to prevent mitochondrial diseases. Hum. Reprod. Update *31*, 269–306. https://doi.org/10.1093/humupd/dmaf004.

151. Stewart, J.B., and Chinnery, P.F. (2015). The dynamics of mitochondrial DNA heteroplasmy: implications for human health and disease. Nat. Rev. Genet. *16*, 530–542. https://doi.org/10.1038/nrg3966.

152. van den Ameele, J., Li, A.Y.Z., Ma, H., and Chinnery, P.F. (2020). Mitochondrial heteroplasmy beyond the oocyte bottleneck. Semin. Cell Dev. Biol. *97*, 156–166. https://doi.org/10.1016/j.semcdb.2019.10.001.

153. Hyslop, L.A., Blakely, E.L., Aushev, M., Marley, J., Takeda, Y., Pyle, A., Moody, E., Feeney, C., Dutton, J., Shaw, C., et al. (2025). Mitochondrial Donation and Preimplantation Genetic Testing for mtDNA Disease. N. Engl. J. Med. *393*, 438–449. https://doi.org/10.1056/NEJMoa2415539.

154. Tachibana, M., Sparman, M., Sritanaudomchai, H., Ma, H., Clepper, L., Woodward, J., Li, Y., Ramsey, C., Kolotushkina, O., and Mitalipov, S. (2009). Mitochondrial gene replacement in primate offspring and embryonic stem cells. Nature *461*, 367–372. https://doi.org/10.1038/nature08368.

155. Lee, H.S., Ma, H., Juanes, R.C., Tachibana, M., Sparman, M., Woodward, J., Ramsey, C., Xu, J., Kang, E.J., Amato, P., et al. (2012). Rapid mitochondrial DNA segregation in primate preimplantation embryos precedes somatic and germline bottleneck. Cell Rep. *1*, 506–515. https://doi.org/10.1016/j.celrep.2012.03.011.

156. Ma, H., Van Dyken, C., Darby, H., Mikhalchenko, A., Marti-Gutierrez, N., Koski, A., Liang, D., Li, Y., Tippner-Hedges, R., Kang, E., et al. (2021). Germline transmission of donor, maternal and paternal mtDNA in primates. Hum. Reprod. *36*, 493–505. https://doi.org/10.1093/humrep/deaa308.

157. Tachibana, M., Amato, P., Sparman, M., Woodward, J., Sanchis, D.M., Ma, H., Gutierrez, N.M., Tippner-Hedges, R., Kang, E., Lee, H.S., et al. (2013). Towards germline gene therapy of inherited mitochondrial diseases. Nature *493*, 627–631. https://doi.org/10.1038/nature11647.

158. Paull, D., Emmanuele, V., Weiss, K.A., Treff, N., Stewart, L., Hua, H., Zimmer, M., Kahler, D.J., Goland, R.S., Noggle, S.A., et al. (2013). Nuclear genome transfer in human oocytes eliminates mitochondrial DNA variants. Nature *493*, 632–637. https://doi.org/10.1038/nature11800.

159. Kang, E., Wu, J., Gutierrez, N.M., Koski, A., Tippner-Hedges, R., Agaronyan, K., Platero-Luengo, A., Martinez-Redondo, P., Ma, H., Lee, Y., et al. (2016). Mitochondrial replacement in human oocytes carrying pathogenic mitochondrial DNA mutations. Nature *540*, 270–275. https://doi.org/10.1038/nature20592.

160. Hudson, G., Takeda, Y., and Herbert, M. (2019). Reversion after replacement of mitochondrial DNA. Nature *574*, E8–E11. https://doi.org/10.1038/s41586-019-1623-3.

161. Costa-Borges, N., Nikitos, E., Späth, K., Miguel-Escalada, I., Ma, H., Rink, K., Coudereau, C., Darby, H., Koski, A., Van Dyken, C., et al. (2023). First pilot study of maternal spindle transfer for the treatment of repeated in vitro fertilization failures in couples with idiopathic infertility. Fertil. Steril. *119*, 964–973. https://doi.org/10.1016/j.fertnstert.2023.02.008.

162. Zhang, J., Liu, H., Luo, S., Lu, Z., Chávez-Badiola, A., Liu, Z., Yang, M., Merhi, Z., Silber, S.J., Munné, S., et al. (2017). Live birth derived from oocyte spindle transfer to prevent mitochondrial disease. Reprod. Biomed. Online *34*, 361–368. https://doi.org/10.1016/j.rbmo.2017.01.013.

163. McGrath, J., and Solter, D. (1983). Nuclear transplantation in the mouse embryo by microsurgery and cell fusion. Science *220*, 1300–1302. https://doi.org/10.1126/science.6857250.

164. Meirelles, F.V., and Smith, L.C. (1997). Mitochondrial genotype segregation in a mouse heteroplasmic lineage produced by embryonic karyoplast transplantation. Genetics *145*, 445–451. https://doi.org/10.1093/genetics/145.2.445.

165. Craven, L., Tuppen, H.A., Greggains, G.D., Harbottle, S.J., Murphy, J.L., Cree, L.M., Murdoch, A.P., Chinnery, P.F., Taylor, R.W., Lightowlers, R.N., et al. (2010). Pronuclear transfer in human embryos to prevent transmission of mitochondrial DNA disease. Nature *465*, 82–85. https://doi.org/10.1038/nature08958.

166. Li, C.Y., Liu, X.C., Li, Y.Z., Wang, Y., Nie, Y.H., Xu, Y.T., Zhang, X.T., Lu, Y., and Sun, Q. (2024). Generation of mitochondrial replacement monkeys by female pronucleus transfer. Zool. Res. *45*, 292–298. https://doi.org/10.24272/j.issn.2095-8137.2023.287.

167. Wu, K., Chen, T., Huang, S., Zhong, C., Yan, J., Zhang, X., Li, J., Gao, Y., Zhao, H., and Chen, Z.J. (2017). Mitochondrial replacement by

pre-pronuclear transfer in human embryos. Cell Res. *27*, 834–837. https://doi.org/10.1038/cr.2017.48.

168. Hyslop, L.A., Blakeley, P., Craven, L., Richardson, J., Fogarty, N.M.E., Fragouli, E., Lamb, M., Wamaitha, S.E., Prathalingam, N., Zhang, Q., et al. (2016). Towards clinical application of pronuclear transfer to prevent mitochondrial DNA disease. Nature *534*, 383–386. https://doi.org/10.1038/nature18303.

169. Fan, X.Y., Guo, L., Chen, L.N., Yin, S., Wen, J., Li, S., Ma, J.Y., Jing, T., Jiang, M.X., Sun, X.H., et al. (2022). Reduction of mtDNA heteroplasmy in mitochondrial replacement therapy by inducing forced mitophagy. Nat. Biomed. Eng. *6*, 339–350. https://doi.org/10.1038/s41551-022-00881-7.

170. McFarland, R., Hyslop, L.A., Feeney, C., Pillai, R.N., Blakely, E.L., Moody, E., Prior, M., Devlin, A., Taylor, R.W., Herbert, M., et al. (2025). Mitochondrial Donation in a Reproductive Care Pathway for mtDNA Disease. N. Engl. J. Med. *393*, 461–468. https://doi.org/10.1056/NEJMoa2503658.

171. Dalton, C.M., and Carroll, J. (2013). Biased inheritance of mitochondria during asymmetric cell division in the mouse oocyte. J. Cell Sci. *126*, 2955–2964. https://doi.org/10.1242/jcs.128744.

172. Wang, T., Sha, H., Ji, D., Zhang, H.L., Chen, D., Cao, Y., and Zhu, J. (2014). Polar body genome transfer for preventing the transmission of inherited mitochondrial diseases. Cell *157*, 1591–1604. https://doi.org/10.1016/j.cell.2014.04.042.

173. Wu, K., Zhong, C., Chen, T., Zhang, X., Tao, W., Zhang, J., Li, H., Zhao, H., Li, J., and Chen, Z.J. (2017). Polar bodies are efficient donors for reconstruction of human embryos for potential mitochondrial replacement therapy. Cell Res. *27*, 1069–1072. https://doi.org/10.1038/cr.2017.67.

174. Ma, H., O'Neil, R.C., Marti Gutierrez, N., Hariharan, M., Zhang, Z.Z., He, Y., Cinnioglu, C., Kayali, R., Kang, E., Lee, Y., et al. (2017). Functional Human Oocytes Generated by Transfer of Polar Body Genomes. Cell Stem Cell *20*, 112–119. https://doi.org/10.1016/j.stem.2016.10.001.

175. Wang, Z., Li, Y., Yang, X., Wang, Y., Nie, Y., Xu, Y., Zhang, X., Lu, Y., Zhang, T., Liu, Q., et al. (2021). Mitochondrial replacement in macaque monkey offspring by first polar body transfer. Cell Res. *31*, 233–236. https://doi.org/10.1038/s41422-020-0381-y.

176. Li, W., Liao, X., Lin, K., Cai, R., Guo, H., Ma, M., Wang, Y., Xie, Y., Zhang, S., Yan, Z., et al. (2023). Earlier second polar body transfer and further mitochondrial carryover removal for potential mitochondrial replacement therapy. MedComm *4*, e217. https://doi.org/10.1002/mco2.217.

177. Dong, L.F., Rohlena, J., Zobalova, R., Nahacka, Z., Rodriguez, A.M., Berridge, M.V., and Neuzil, J. (2023). Mitochondria on the move: Horizontal mitochondrial transfer in disease and health. J. Cell Biol. *222*, e202211044. https://doi.org/10.1083/jcb.202211044.

178. Li, M., Wu, L., Si, H., Wu, Y., Liu, Y., Zeng, Y., and Shen, B. (2025). Engineered mitochondria in diseases: mechanisms, strategies, and applications. Signal Transduct. Target. Ther. *10*, 71. https://doi.org/10.1038/s41392-024-02081-y.

179. Lin, R.Z., Im, G.B., Luo, A.C., Zhu, Y., Hong, X., Neumeyer, J., Tang, H.W., Perrimon, N., and Melero-Martin, J.M. (2024). Mitochondrial transfer mediates endothelial cell engraftment through mitophagy. Nature *629*, 660–668. https://doi.org/10.1038/s41586-024-07340-0.

180. Jacoby, E., Ben Yakir-Blumkin, M., Blumenfeld-Kan, S., Brody, Y., Meir, A., Melamed-Book, N., Napso, T., Pozner, G., Saadi, E., Shabtay-Orbach, A., et al. (2021). Mitochondrial augmentation of CD34(+) cells from healthy donors and patients with mitochondrial DNA disorders confers functional benefit. npj Regen. Med. *6*, 58. https://doi.org/10.1038/s41536-021-00167-7.

181. Borcherding, N., and Brestoff, J.R. (2023). The power and potential of mitochondria transfer. Nature *623*, 283–291. https://doi.org/10.1038/s41586-023-06537-z.

Cell Reports Medicine, Volume *7*

Supplemental information

Emerging therapeutic strategies for mitochondrial DNA-related diseases

Rubing Shi, Micol Falabella, Jana Aref, Michael G. Hanna, Michal Minczuk, Carlo Viscomi, and Robert D.S. Pitceathly

Supplementary Table 1. Preclinical *in vitro* studies of gene therapy for PMDs

Gene (variant)	Disease	Model	Results	Refs
**Allotopic expression**				
MT-ND6 (m.14484T>C)	LHON	cybrids	Complex I restoration	17
**mitoREs**				
MT-ATP6 (m.8993T>G)	NARP, MILS	cybrids	↓ mutant mtDNA, restoration of ATP and membrane potential	64
MT-ATP6 (m.8993T>G)	NARP, MILS	cybrids	↓ mutant mtDNA, improved ATP synthase, ↓ lactate	65
**mtZFNs**				
MT-ATP6 (m.8993T>G)	NARP, MILS	cybrids	↓ mutant mtDNA	19
MT-ATP6 (m.8993T>G)	NARP, MILS	cybrids	↓ mutant mtDNA	20
CD (m.8483_13459del4977)	CPEO, KSS, PMPS	cybrids	↓ mutant mtDNA, restoration of MRF	
MT-ATP6 (m.8993T>G)	NARP, MILS	cybrids	↓ mutant mtDNA, restoration of MRF	69
MT-ATP6 (m.8993T>G)	NARP, MILS	cybrids	↓ mutant mtDNA, restoration of MRF	70
**mitoTALENs**				
SLSMD (m.8483_13459del)	CPEO, KSS, PMPS	cybrids	↓ mtDNA deletions and point mutations	21
MT-ND6 (m.14459G>A)	LHON	cybrids	↓ mtDNA deletions and point mutations	
MT-ND6 (m.14459G>A)	LHON	oocytes	↓ mutant mtDNA	75
MT-ATP6 (m.9176T>C)	NARP	oocytes		
MT-TK (m.8344A>G)	MERRF	cybrids	↓ mutant mtDNA, restoration of MRF	76
MT-ND5 (m.13513G>A)	MELAS, LS	cybrids		
MT-TL1 (m.3243A>G)	MELAS	iPSCs, porcine oocytes	↓ mutant mtDNA, restoration of MRF	77
MT-ND5 (m.13513G>A)	MELAS, LS	iPSCs	↓ mutant mtDNA	79
MT-TL1 (m.3243A>G)	MELAS	iPSCs	↓ mutant mtDNA	80
MT-ND5 (m.13513G>A)	MELAS, LS	iPSCs	Reduction mutant mtDNA	83
**mitoTev-TALE**				
MT-TK (m.8344A>G)	MERRF	cybrids	↓ mutant mtDNA, restoration of MRF	78
**mitoARCUS**				
MT-TA (m.5024C>T)	CM	MEFs	↓ mutant mtDNA	23
MT-TL1 (m.3243A>G)	MELAS	cybrids	↓ mutant mtDNA, improved MRF	24
**DdCBEs**				
MT-TI (m.4291T>C)	Gitelman-like syndrome	fibroblasts	↓ mutant mtDNA, rescued MRF	31
MT-TI (m.4300A>G)	HCM	iPSCs	↓ mutant mtDNA, rescued MRF	87
MT-TA (m.5024C>T)	CM	MEFs	restored mitochondrial $tRNA^{Ala}$ level	26
**mitoABE**				
MT-ND4 (m.11778G>A)	LHON	Patient cells	↓ mutant mtDNA, rescued MRF	29
**TALED**				
MT-ND4 (m.11778G>A)	LHON	MEFs	Mutation recovery ratio ~8%, rescued MRF	109

Table S1. Preclinical *in vitro* studies of gene therapy for PMDs. Related to Table 1.
Abbreviations: ATP, adenosine triphosphate; CM, cardiomyopathy; CPEO, chronic progressive external ophthalmoplegia; DdCBEs, DddA-derived cytosine base editors; HCM, hypertrophic cardiomyopathy; iPSCs, induced pluripotent stem cells; KSS, Kearns-Sayre syndrome; LHON, Leber hereditary optic neuropathy; LS, Leigh syndrome; MEFs, mouse embryonic fibroblasts; MELAS, mitochondrial encephalopathy, lactic acidosis and stroke-like episodes; MERRF, myoclonic epilepsy with ragged red fibres; MILS, maternally inherited Leigh syndrome; mitoABE, mitochondria-targeted adenine base editor; mitoREs, mitochondria-targeted restriction endonucleases; mitoTALENs, mitochondria-targeted transcription activator–like effector nucleases; MRF, mitochondrial respiratory function; MT-ATP6, mitochondrially encoded ATP synthase membrane subunit 6; mtDNA, mitochondrial DNA; MT-ND4/5/6, mitochondrially encoded NADH dehydrogenase subunits 4, 5, and 6; MT-TA, mitochondrially encoded tRNA-Ala; MT-TI, mitochondrially encoded tRNA-Ile; MT-TK, mitochondrially encoded tRNA-Lys; MT-TL1, mitochondrially encoded tRNA-Leu; mtZFN, mitochondria-targeted zinc finger nuclease; NARP, neuropathy, ataxia, and retinitis pigmentosa; PMPS, Pearson's marrow-pancreas syndrome; SLSMD, single large-scale mitochondrial DNA deletion syndromes; TALEDs, TALE-linked deaminases.

Supplementary Table 2. Preclinical *in vivo* studies of gene therapy for PMDs

Gene (variant)	Disease	Model	Vector	AR	Target organ	Results	Refs
**mitoREs**							
-	-	NZB/BALB	rAd5/AAV1/2	IC/IM	Brain, SM	efficient shift in mtDNA heteroplasmy	66
-	-	NZB/BALB	AAV9	IP/TV	SM, heart	efficient shift in mtDNA heteroplasmy	67
**mitoZFNs**							
MT-TA (m.5024C>T)	CM	mouse	AAV9.45	IV	Heart	↓ mutant mtDNA, increased stability of $tRNA^{Ala}$, ↓ glycolysis, improved mitochondrial respiration	71
MT-TA (m.5024C>T)	CM	mouse	AAV9.45/AAV9	IM/IV	SM, heart	↓ mutant mtDNA, increased stability of $tRNA^{Ala}$ with minimized immune responses	68
**mitoTALENs**							
-	-	NZB/BALB embryos	-	-	Brain, SM, heart, liver	efficient shift in mtDNA heteroplasmy	75
MT-TA (m.5024C>T)	CM	mouse	AAV9	IM/IV/IP	SM, heart	↓ mutant mtDNA and restored mitochondrial $tRNA^{Ala}$ level	22
**mitoARCUS**							
MT-TA (m.5024C>T)	CM	mouse	AAV9	RO	SM, heart, liver	↓ mutant mtDNA and restored mitochondrial $tRNA^{Ala}$ level	23
MT-TL1 (m.3243A>G)	MELAS	mouse	AAV9	RO	xenograft	↓ mutant mtDNA	24
**DdCBEs**							
MT-TA (m.5024C>T)	CM	mouse	AAV9	RO	SM, heart	restored mitochondrial tRNAAla level and reversed lactate amounts	26
**TALEDs**							
MT-ND4 (m.11778G>A)	LHON	mouse	AAV2	IVT	eye	↓ mutant mtDNA and restored retinal ganglion cell function	109

Table S2. Preclinical *in vivo* studies of gene therapy for PMDs. Related to Table 1.
Abbreviations: AR, administration route; CM, cardiomyopathy; DdCBEs, DddA-derived cytosine base editors; IC, intracerebral; IM, intramuscular; IP, intraperitoneal; iPSCs, induced pluripotent stem cells; IV, intravenous; IVT, intravitreal; LHON, Leber hereditary optic neuropathy; MELAS, mitochondrial encephalopathy, lactic acidosis and stroke-like episodes; mitoABE, mitochondria-targeted adenine base editor; mitoREs, mitochondria-targeted restriction endonucleases; mitoTALENs, mitochondria-targeted transcription activator–like effector nucleases; mtDNA, mitochondrial DNA; MT-TA, mitochondrially encoded tRNA-Ala; MT-TL1, mitochondrially encoded tRNA-Leu; mtZFN, mitochondria-targeted zinc finger nuclease; NZB/BALB, New Zealand Black × BALB/c hybrid mice RO, retro-orbital; SM, skeletal muscle; TALEDs, TALE-linked deaminases; TV, temporal vein.
